# Supplementary material for: Cohort profile: Scotland’s record-linkage e-cohorts of people with intellectual disabilities, and autistic people (SCIDA)
Source: BMJ Open. 2022 May 12;12(5):e057230. doi: 10.1136/bmjopen-2021-057230 (PMC9109103; doi:10.1136/bmjopen-2021-057230)
Supplement: Supplementary data [file bmjopen-2021-057230supp001.pdf]

Supplementary file 1. List of variables included in the two datasets

DATASET 1

Type of variable	Outcome	Exposure	Potential confounding factors
**Scotland's Census 2011**			
Read-through Index number for linkage		N/A	N/A
Month and year of birth			X
Living arrangement - seven exclusive values			X
Scottish Index of Multiple Deprivation 2012			X
Urban-rural classification 8-fold 2011-12			X
Heath Board area 2011			X
Council area 2011			X
Long-term health condition: autism		X	
Long-term health condition: learning disability		X	
Sex			X
**NRS Death register**			
CHI		N/A	N/A
Age at death	X		
Main cause of death	X		
All causes of death (1-11)	X		
Date of death	X		
Scottish Index of Multiple Deprivation 2012			X
Place of death			X
**SMR06**			
CHI		N/A	N/A
Date of incidence	X		
Date of registration	X		
Death certificate only	X		
Side			X
Site (ICD09/ICD10/ICD02)	X		
Type ICD0/ICD02/ICD03	X		
Treated with surgery			X
Treated with radiotherapy			X
Treated with chemotherapy			X
Treated with hormones			X
Treated with other therapy			X

DATASET 2

Type of variable	Outcome	Exposure	Potential confounding factors
**Scotland's Census 2011**			
Read-through Index number for Census 2011		N/A	N/A
Month and year of birth			X
Living arrangements - seven exclusive values			X
Scottish Index of Multiple Deprivation 2012			X
Urban-rural classification 8-fold 2011-12			X
Heath Board area 2011			X
Council area 2011			X
Activity last week			X
Adults in employment, number in household			X
Adults in household, number of			X
Carers in household with economic activity			X
Provision of unpaid care			X
Cars or vans, number of			X
Central heating type in household			X
Unpaid carers in household, number of			X
Limitation of activities			X
Distance travelled to work or place of study			X
Recorded ethnicity			X
Nature of communal establishment			X
Ever employed			X
Number of residents in family			X
Family status			X
Count of dependent children in family			X
General Health status			X
Highest level of qualification			X
Hours worked (grouped)			X
Language proficiency			X
Marital status			X
Long-term health condition: blind		X	X
Long-term health condition: autism		X	X
Long-term health condition: deaf		X	X
Long-term health condition: dyslexia		X	X
Long-term health condition: learning disability		X	X
Long-term health condition: mental health condition		X	X
Long-term health condition: physical disability		X	X

**Type of variable**	**Outcome**	**Exposure**	**Potential confounding factors**
Occupancy rating			X
Occupancy status			X
Parent to household member			X
Population density of datazone			X
Persons per room, number in household			X
Sex			X
Household size			X
Students living away from home during term time (aged 4 and over)			X
Schoolchild or full-time student indicator			X
Tenure of household			X
Term-time address indicator			X
Accommodation type			X
**NRS Death register**			
CHI		N/A	N/A
Age at death	X		
Main cause of death	X		
All causes of death (1-11)	X		
Month of death	X		
Scottish Index of Multiple Deprivation 2012			X
Place of death			X
**Prescribing Information System**			
CHI		N/A	N/A
Date medication prescribed			X
Date medication dispensed			X
Date medication paid			X
BNF codes of drugs for: asthma/COPD; angina/congestive heart failure/hypertension; peptic ulcer/reflux; constipation; diabetes; thyroid dysfunction; depression; bipolar disorder; anxiety/sleep; psychosis; ADHD; epilepsy; glaucoma		X	X
BNF root drug		X	
Prescribed item name/approved name		X	
Prescribed item strength			X
Number of defined daily doses dispensed			X
Number of dispensed/paid items			X
Dispensed/paid quantity			X
**SMR01**			
CHI		N/A	N/A
Date of admission	X		
Date of discharge	X		
Main condition on discharge	X		

**Type of variable**	**Outcome**	**Exposure**	**Potential confounding factors**
Secondary condition on discharge (1-5)	X		
Main operation	X		
Other operations (1-3)	X		
Length of stay	X		
CIS marker	X		
Inpatient/day case marker	X		
Type of facility/management patient			X
Type of admission	X		
Admission reason	X		
**SMR04**			
CHI		N/A	N/A
Date of admission	X		
Date of discharge	X		
Main condition on discharge	X		
Secondary condition on discharge (1-5)	X		
Main operation	X		
Other operations (1-3)	X		
Main condition on admission	X		
Other conditions on admission (1-3)	X		
Previous psychiatric care			X
Type of psychiatric care			X
**SCI-Diabetes**			
CHI		N/A	N/A
Date of diagnosis			X
Type of diabetes		X	
HbA1c	X		
Hypoglycaemic coma	X		
Attendance at hospital diabetes clinic	X		
Arrangements for formal diabetes care	X		
Drugs			X
Date of death	X		
Cause of death	X		
Underlying cause of death	X		
Weight			X
Height			X
Body Mass Index			X
Systolic bp			X
Diastolic bp			X
Smoking status at date of contact			X
Year patient stopped smoking			X
Alcohol intake			X
Diabetic review on this date	X		
Referral to defined health care professional	X		
Seen by defined health care professional	X		

Type of variable	Outcome	Exposure	Potential confounding factors
**National Bowel Screening programme**			
CHI		N/A	N/A
Health board of residence			X
Date screening test kit sent			X
Screening test result	X		
Colonoscopy performed?	X		
Reason for not having a colonoscopy	X		
Colonoscopy completed	X		
Invasive cancer detected?	X		
ICD 10 classification of neoplasm	X		
Complication arising from colonoscopy			X
TNM derived Dukes' stage	X		
Polyps detected	X		
Adenoma detected	X		
**Cervical Cancer screening**			
CHI		N/A	N/A
Date of test result	X		
SIMD 2012 deprivation category			X
Date of HPV Immunisation	X		
HPV Immunisation Status	X		
Screened within 3.5yr	X		
Screened within 5.5yr	X		
