# Supplementary material for: Cohort profile: Scotland’s record-linkage e-cohorts of people with intellectual disabilities, and autistic people (SCIDA)
Source: BMJ Open. 2022 May 12;12(5):e057230. doi: 10.1136/bmjopen-2021-057230 (PMC9109103; doi:10.1136/bmjopen-2021-057230)
Supplement: Supplementary data [file bmjopen-2021-057230supp002.pdf]

Supplementary file 2. Data processing flowchart

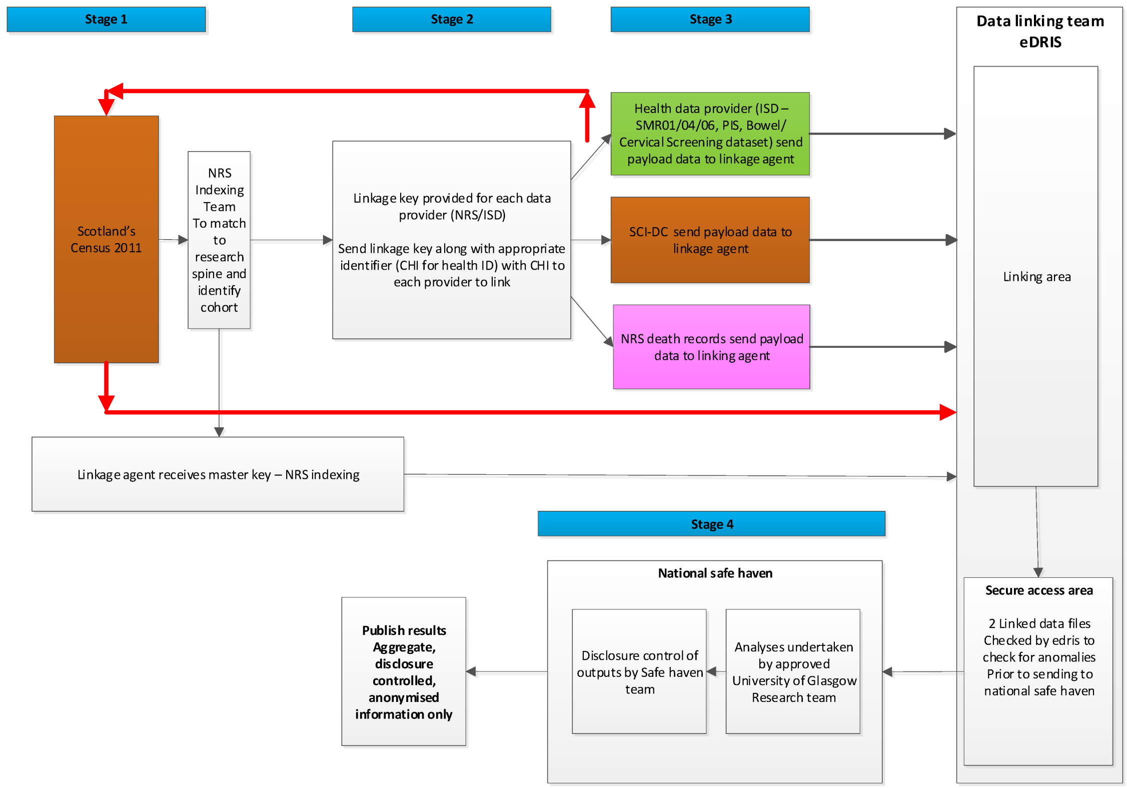

CHI=community health index; eDRIS=electronic data research and innovation service; ISD=information services division of national services Scotland; NRS=national records of Scotland; PIS=prescribing information system; SCI-DC=Scottish care information–diabetes collaboration; SMR=Scottish morbidity record
