# Supplementary material for: Cohort profile: Scotland’s record-linkage e-cohorts of people with intellectual disabilities, and autistic people (SCIDA)
Source: BMJ Open. 2022 May 12;12(5):e057230. doi: 10.1136/bmjopen-2021-057230 (PMC9109103; doi:10.1136/bmjopen-2021-057230)
Supplement: Author's manuscript [file bmjopen-2021-057230.draft_revisions.pdf]

BMJ Open is committed to open peer review. As part of this commitment we make the peer review history of every article we publish publicly available.

When an article is published we post the peer reviewers' comments and the authors' responses online. We also post the versions of the paper that were used during peer review. These are the versions that the peer review comments apply to.

The versions of the paper that follow are the versions that were submitted during the peer review process. They are not the versions of record or the final published versions. They should not be cited or distributed as the published version of this manuscript.

BMJ Open is an open access journal and the full, final, typeset and author-corrected version of record of the manuscript is available on our site with no access controls, subscription charges or pay-per-view fees (http://bmjopen.bmj.com).

If you have any questions on BMJ Open's open peer review process please email info.bmjopen@bmj.com

Cohort profile: Scotland's record-linkage e-cohorts of people with intellectual disabilities, and autistic people (SCIDA)

Journal:	*BMJ Open*
Manuscript ID	bmjopen-2021-057230
Article Type:	Cohort profile
Date Submitted by the Author:	13-Sep-2021

Complete List of Authors:	Cooper, Sally-Ann; University of Glasgow College of Medical Veterinary and Life Sciences, Institute of Health and Wellbeing<br>Henderson, Angela; University of Glasgow, Institute of Health and Wellbeing<br>Kinnear, Deborah; University of Glasgow, Institute of Health and Wellbeing<br>Mackay, Daniel; University of Glasgow,<br>Fleming, Michael ; University of Glasgow, Public Health<br>Smith, Gillian; University of Glasgow Institute of Health and Wellbeing, Mental Health and Wellbeing<br>Hughes-McCormack, Laura; University of Glasgow, Institute of Health and Wellbeing<br>Rydzewska, Ewelina; University of Glasgow, Institute of Health and Wellbeing<br>Dunn, Kirsty; University of Glasgow, Institute of Health and Wellbeing<br>Pell, J. P.; Univ Glasgow<br>Melville, Craig; University of Glasgow

Keywords:	EPIDEMIOLOGY, Quality in health care < HEALTH SERVICES ADMINISTRATION & MANAGEMENT, MENTAL HEALTH, PUBLIC HEALTH

SCHOLARONE™ Manuscripts

I, the Submitting Author has the right to grant and does grant on behalf of all authors of the Work (as defined in the below author licence), an exclusive licence and/or a non-exclusive licence for contributions from authors who are: i) UK Crown employees; ii) where BMJ has agreed a CC-BY licence shall apply, and/or iii) in accordance with the terms applicable for US Federal Government officers or employees acting as part of their official duties; on a worldwide, perpetual, irrevocable, royalty-free basis to BMJ Publishing Group Ltd ("BMJ") its licensees and where the relevant Journal is co-owned by BMJ to the co-owners of the Journal, to publish the Work in this journal and any other BMJ products and to exploit all rights, as set out in our licence.

The Submitting Author accepts and understands that any supply made under these terms is made by BMJ to the Submitting Author unless you are acting as an employee on behalf of your employer or a postgraduate student of an affiliated institution which is paying any applicable article publishing charge ("APC") for Open Access articles. Where the Submitting Author wishes to make the Work available on an Open Access basis (and intends to pay the relevant APC), the terms of reuse of such Open Access shall be governed by a Creative Commons licence – details of these licences and which Creative Commons licence will apply to this Work are set out in our licence referred to above.

Other than as permitted in any relevant BMJ Author's Self Archiving Policies, I confirm this Work has not been accepted for publication elsewhere, is not being considered for publication elsewhere and does not duplicate material already published. I confirm all authors consent to publication of this Work and authorise the granting of this licence.

For peer review only

Cohort profile: Scotland's record-linkage e-cohorts of people with intellectual disabilities, and autistic people (SCIDA)

***Sally-Ann Cooper**
Angela Henderson
Deborah Kinnear
Daniel Mackay
Michael Fleming
Gillian Smith
Laura Anne Hughes-McCormack
Ewelina Rydzewska
Kirsty Dunn
J. P. Pell
Craig Melville

*Corresponding author:
Sally-Ann Cooper
University of Glasgow, Institute of Health and Wellbeing,
1st Floor Administrative Building, Gartnavel Royal Hospital, 1,055 Great Western Road, Glasgow, UK
Sally-Ann.Cooper@glasgow.ac.uk

For peer review only

For peer review only - http://bmjopen.bmj.com/site/about/guidelines.xhtml

Abstract

Purpose

To investigate health, mortality, and health-care inequalities experienced by people with intellectual disabilities, and autistic people, and their determinants. This is an important step towards identifying and implementing solutions to reduce inequalities.

Participants

Scotland's Census, 2011 was used to identify Scotland's citizens with intellectual disabilities, and autistic citizens, and representative general population samples with neither. Using Scotland's community health index, the Census data (demography, household, employment, long-term conditions) were linked for each individual with routinely collected health, death, and health-care data: Scotland's register of deaths, Scottish morbidity data 06 (SMR06: cancer incidence, mortality, treatments), Prescribing Information System (identifying asthma/COPD; angina/congestive heart failure/hypertension; peptic ulcer/reflux; constipation; diabetes; thyroid disorder; depression; bipolar disorders; anxiety/sleep; psychosis; attention deficit hyperactivity disorder; epilepsy; glaucoma), SMR01 (general/acute hospital admissions and causes, ambulatory care sensitive admissions), SMR04 (mental health admissions and causes), Scottish Care Information–Diabetes Collaboration (diabetic care quality, diabetic outcomes), national bowel screening programme, and cervical screening.

Findings to date

Of the whole population, 0.5% had intellectual disabilities, and 0.6% were autistic. Linkage was successful for >92%. The resultant e-cohorts include: (1) 22,538 people with intellectual disabilities (12,837 male, 9,701 female), 4,509 of whom are children <16 years, (2) 27,741 autistic people (21,390 male, 6,351 female), 15,387 of whom are children <16 years, and (3) representative general population samples with neither condition. Very good general health was reported for only 3,389 (15.0%) people with intellectual disabilities, and 10,510 (38.0%) autistic people, compared with 52.4% of the general population. Mental health conditions were reported for 4,755 (21.1%) people with intellectual disabilities, and 3,998 (14.4%) autistic people, compared with 4.2% of the general population.

Future plans

Analyses will determine the extent of premature mortality, causes of death, and avoidable deaths, profile of health conditions and cancers, health-care quality and screening, and determinants of mortality and health-care.

Strengths and limitations of this study

- 94% Census completion for a whole country population, and >92% successful record linkage.
- Record linkage across multiple data sources.
- Limitations include the use of routinely collected data.
- Reliance upon recorded cause of death on death certificates.
- Self/proxy reporting of intellectual disabilities and autism, and other Census data.

For peer review only - http://bmjopen.bmj.com/site/about/guidelines.xhtml

Introduction

People with intellectual disabilities are thought to die about 20 years earlier than other people,[1] or 28 years earlier for people with Down syndrome,[2] but most studies are small. A large English study found a standardised mortality ratio (SMR) of 3.18 (95% confidence intervals 2.94, 3.43).[3] Two large Scottish studies reported an SMR of 2.24 (1.98, 2.49) in adults overall; 5.28 (3.98, 6.57) for Down syndrome adults, and 1.93 (1.68, 2.18) for adults without Down syndrome,[4] and an SMR of 11.6 (9.6, 14.0) in children overall.[5] Autism is also thought to be associated with premature death, though there have been few studies, and co-occurring intellectual disabilities may account for some of the reported findings.[6-9] The commonest causes of death of people with intellectual disabilities are likely to differ from other people. For example, aspiration pneumonia which is avoidable, may occur quite commonly,[3,4,10] and SMRs have been reported as 2.4 (1.3, 3.8) for colorectal cancer, and 2.3 (1.0, 4.3) for female genital cancers[3]. Additionally, probably about 40% of early mortality would have been amenable to good quality health care, which may be double the amount that occurs in other people.[4,10,11] Causes of death of autistic people may also differ from those in the general population, with deaths related to the nervous system, and injury and poisoning reported to be the most common causes.[8] These inequality are a major injustice that need addressing. An important step towards this is to gain a better understanding of health, mortality, health-care, and their determinants in people with intellectual disabilities and autistic people.

The health profiles of people with intellectual disabilities and autistic people differ from other people, which has a bearing on mortality. People with intellectual disabilities have, for example, high rates of epilepsy, mental illness, gastrointestinal disease, antipsychotic use, and polypharmacy.[12-14] They may have higher diabetes rates,[15,16] due to sedentary lifestyles, obesity, and antipsychotics.[17,18] Cancer profiles appear to differ from other people, with colorectal, stomach, oesophageal, brain, and uterine cancer, and leukaemia all having been reported to have higher incidence in people with intellectual disabilities, but with some inconsistencies between studies, and wide confidence intervals due to study sizes.[19-21] Higher incidence of gastrointestinal cancers is plausible, due to poor diets, lifestyle, and high rates of helicobacter pylori and gastro-oesophageal reflux disorder, and lower screening uptake. Multi-morbidity is typical in people with intellectual disabilities,[13,14] and poor health is reported for people with intellectual disabilities across the full life-course.[22] In autistic people, general health is also reported to be poor across the lifespan.[23,24] Autistic people commonly have co-morbid physical conditions, and some are more prevalent than in the general population, including sleep problems, epilepsy, sensory impairments, atopy, autoimmune disorders, and obesity, but not asthma.[25] However, there are substantial gaps in the evidence-base on the health of autistic people, and some findings are inconsistent.[25]

Despite this higher burden of health need and early mortality, health-care has been reported to be poorer for people with intellectual disabilities,[26] and for autistic people,[27] and barriers in access to health-care have been reported.[26,27] Adults with intellectual disabilities are reported to participate less in cervical screening,[28] and bowel screening[29] than other people. There is also some indication that people with intellectual disabilities and autistic people are more likely to be hospitalised for ambulatory care-sensitive conditions than other people; these are conditions that should be fully manageable in primary health care settings, and so such hospitalisations are a further marker for poor care.[30,31] These factors may impact on poorer outcomes and survival, due to later stage disease when treatment is initiated, and poorer management of disease.

For peer review only - http://bmjopen.bmj.com/site/about/guidelines.xhtml

Record-linkage of Scotland's Census, 2011 to Scotland's other rich routinely collected health datasets and national statistics provides an excellent vehicle for detailed investigation of these issues. Scotland's Census, 2011 systematically questioned whether every person in the country had intellectual disabilities, and/or autism, and distinguished intellectual disabilities from dyslexia and other specific learning disabilities. It included data on 94% of the whole Scottish population, and imputed the remaining 6% (5,295,403 people). Due to its large size, it has power, when linked to other databases to answer important questions on health, causes of mortality, determinants, and health-care in people with intellectual disabilities, and autistic people. This is an important step leading towards to a reduction in morbidity, early mortality and avoidable deaths, and improvements in health-care of people with intellectual disabilities and autistic people.

In Scotland's Census, 2011, of the whole Scottish population, 0.5% were recorded as having intellectual disabilities, and 0.6% were recorded as being autistic people. Regarding autism, 1.6% of all Scottish children and young people (<25 years of age) were recorded to have autism, whilst 0.2% of adults aged 25 years and over were recorded to have autism, reflecting the diagnostic broadening of autism spectrum disorders in recent years, and greater awareness of the condition.

We created two datasets, with record linkage to several of Scotland's health datasets. Linkage was undertaken for the 94% of the Scottish population who completed the Scottish Census, 2011. Of the people with intellectual disabilities recorded in Scotland's Census, 2011, 22,538 (92.9%) were successfully linked with their health records. Of the people with autism recorded in Scotland's Census, 2011, 27,741 (94.6%) were successfully linked with their health records. Regarding the general population who had neither intellectual disabilities nor autism, of the 15% randomly selected, 700,437 (95.1%) were successfully linked to their health records; whilst of the 5% randomly selected, 233,378 (95.1%) were successfully linked to their health records. Hence, each dataset includes data on 22,538 people with intellectual disabilities, 27,741 autistic people, and a randomly selected comparison general population with neither intellectual disabilities nor autism. For ethical reasons, two datasets were necessary to ensure data minimisation, as our research questions on cancer incidence and mortality required a larger comparison group to reach adequate power, due to lower incidence, than was required to answer the other research questions.

- Dataset 1 includes 22,538 people with intellectual disabilities, 27,741 autistic people, and a random sample of 700,437 (15%) of the Scottish population. It will be used to answer research questions on SMRs, causes of deaths, amenable deaths, cancer standardised incidence ratios (SIRs), and cancer SMRs.
- Dataset 2 includes 22,538 people with intellectual disabilities, 27,741 autistic people, and a random sample of 233,378 (5%) of the Scottish population. It will be used to answer research questions on independent predictors of deaths and amenable deaths, comparison of uptake of cervical screening and bowel screening, descriptions of population health burden, and quality of health-care.

The sample size for the comparison sample in dataset 1 was determined using the Scottish crude annual incidence rates and mortality rates for bowel cancer, oesophageal cancer, and uterine cancer as examples, and previously published SIRs and SMRs for people with intellectual disabilities[3,19,21]. The figure shows examples of some of the power graphs produced which led to the decision to use 15% of the Scottish population as the comparison group.

- Insert the figure about here -

For peer review only - http://bmjopen.bmj.com/site/about/guidelines.xhtml

The datasets we linked together were based on these specific research questions posed on (a) people with intellectual disabilities, and (b) autistic people, compared with other people:

1. What are the age and sex-standardised mortality ratios?
2. What are the common causes of death, and what proportion are amenable deaths?
3. Do cancer incidence and mortality differ?
4. What personal (including diseases identified via medication and admissions), household, and service-related factors (including hospital admissions) are markers/predictors of deaths and amenable deaths?
5. Do uptake of the national bowel and cervical cancer screening programmes differ?
6. What is the population health burden (identified via medication and admissions)?
7. Do people with diabetes* or other ambulatory care-sensitive conditions, receive as good health-care, and are diabetes or other ambulatory care-sensitive conditions more likely to result in hospital admissions and deaths?

*Diabetes was selected as an exemplar due to Scotland's Register of Diabetes Care.

Cohort description

Table 1 shows the data sources which were linked, the dates of the data collection, the intended research purposes for the data, and the data controllers.

- Insert table 1 about here -

The variables we selected to include from each of the data sources were outcomes, exposures, or potential confounders. A table of the full list of variables is included in supplementary file 1. Data linkage of Scotland's Census, 2011, and de-identification, were carried out by the National Records of Scotland (NRS) Indexing Service (a Trusted Third Party Linkage Service) to the NRS Indexing Spine, which includes each person's Community Health Index. The Community Health Index is the unique NHS identifier given to everyone in Scotland; hence this indexing then enabled linkage to all the other selected health databases. Supplementary file 2 shows the process. The linked datasets are held in Scotland's national safe-haven, where access is provided to the approved members of our team.

Scotland's Census, 2011 includes information on people staying in Scotland on 27 March 2011. It includes people in both private households and communal establishments (e.g. care homes). One householder on behalf of all occupants in private households, or the manager on behalf of all occupants of communal dwellings, was required to complete the Census questionnaire. Non-responders were followed up, and help was provided to complete the form if needed. It was a legal requirement to complete the Census questionnaire, and a statement on the form said that non-completion, or supplying false information could attract a £1,000 fine. It was estimated to have achieved a 94% response rate.[32] During the original data processing, the Census team adjusted for the 6% for whom data were missing, using a Census Coverage Survey (including around 40,000 households) to estimate numbers and characteristics. Self/proxy-reporting was used to identify people with autism and other long-term conditions from the Census question 20: 'Do you have any of the following conditions which have lasted, or are expected to last, at least 12 months? Tick all that apply'. Respondents were given a choice of 10 response options: (1) deafness or partial hearing loss, (2) blindness or partial sight loss, (3) learning disability (for example, Down's syndrome), (4) learning difficulty (for example, dyslexia), (5) developmental disorder (for example, autistic spectrum disorder or Asperger's syndrome), (6) physical disability, (7) mental health condition, (8) long-term illness, disease or condition (9) other condition, (10) no condition.

For peer review only - http://bmjopen.bmj.com/site/about/guidelines.xhtml

Cognitive question testing with retrospective probing was undertaken on question 20 prior to the Census, to test whether the questions were answered accurately and willingly by respondents, and to identify what changes might be required to improve data quality and/or the acceptability of the response options. The pilot testing was conducted with 102 participants with a mix of gender and age, both with and without health conditions/disabilities (including people with more than one of the conditions), and included people with autism, intellectual disabilities, dyslexia, dyspraxia, speech impairment, mental health conditions (both milder and more serious), and other long-term conditions. This resulted in a redesign of the question on autism, to the version included in the actual Census, to accurately capture the data on autism. Importantly, question 20 distinguished autism from learning disability (which in the UK is synonymous to the international term 'intellectual disabilities'), learning difficulty (which in the UK is synonymous to the international term 'specific learning disability' such as dyslexia), and mental health conditions. All people with a positive response to question 20 on intellectual disabilities and/or autism were included in our record-linked datasets.

The data sources linked to the variables from Scotland's Census, 2011 are described below. All provide routinely collected data for the whole of Scotland. The Scottish Morbidity Record (SMR) routinely records information from episodes of health care.

1. SMR 01 provides data on general/acute hospital admissions and causes using the international statistical classification of diseases and related health problems, 10th edition (ICD-10) codes,[33]
2. SMR 04 provides data on mental health admissions and causes also using ICD-10 codes,
3. SMR 06 is the Cancer Registry and provides information on the disease, its treatments, and outcomes.
4. Prescribing Information System provides information on all medicines that are prescribed and dispensed in the community in Scotland, or issued in hospitals and dispensed in the community, and prescriptions written in Scotland that were dispensed elsewhere in the UK, using British National Formulary codes.
5. Scottish Care Information–Diabetes Collaboration is a national diabetes register that collects data on the quality of care, and outcomes for all patients with diabetes.
6. The national bowel, and cervical screening programmes collect data on screening uptake.
7. Scotland's national death register collates information from death certificates including cause of death as recorded by the certifying clinician, using ICD-10 codes.

Table 2 reports the characteristics of the cohorts with intellectual disabilities, and autism, and the random samples of the general population with neither of these conditions.

- Insert table 2 about here –

Patient and public involvement

This resource and the programme of research it will support, was designed to respond to the growing concern expressed by people with intellectual disabilities, autistic people, their families, and third sector organisations about premature deaths, and poor health-care. The Scottish Learning Disabilities Observatory, where this research is being undertaken, has a specific remit for such work. Its steering group includes partners from third sector organisations, including Down syndrome Scotland, and people with intellectual disabilities, who approved the work plan for this project prior to it commencing.

Findings to date

For peer review only - http://bmjopen.bmj.com/site/about/guidelines.xhtml

To date, research has been completed on the cross-sectional Scottish Census, 2011 data only.

In comparison with the general population, in children/youth, both intellectual disabilities [Odds ratio: OR=18.34 (917.17, 19.58)], and autism [OR=8.40 (8.02, 8.80)] were independently associated with poor general health. In adults, both intellectual disabilities [OR=7.54 (7.02, 8.10)], and autism [OR=4.46 (4.06, 4.89)] were also independently associated with poor general health.[34] People with co-occurring intellectual disabilities and autism combined were particularly vulnerable to poor general health.[35]

In comparison with the general population, mental health conditions were also more common in people with intellectual disabilities [OR=7.1 (6.8, 7.3)],[36] autistic adults [OR=8.6 (8.2, 9.0)],[37] and people with co-occurring intellectual disabilities and autism [OR=130.8 (117.1, 146.1)].[38] Poor general health was also associated with poor mental health in people with intellectual disabilities.[36]

Visual impairment, hearing impairment, and physical disability were also more common than in the general population. For autistic adults, and autistic children/youth respectively, visual impairment [OR=8.5 (7.9, 9.2) and OR=8.9 (8.1-9.7)]; hearing impairment [OR=3.3 (3.1, 3.6) and OR=5.4 (5.1-5.6)]; and physical disability [OR=6.2 (5.8, 6.6) and OR=15.8 (14.1-17.8)] were considerably more common.[37,39] For people with both intellectual disabilities and autism, visual impairment [OR=65.9 (95% CI 58.7, 73.9)]; hearing impairment [OR=22.0 (95% CI 19.2, 25.2)]; and physical disability [OR=157.5 (95%=CI 144.6, 171.7)] were even more common.[37]

Raising awareness of these issues is important, as these conditions are disabling, and in some cases life-limiting if left untreated. In addition to the communication needs that people with intellectual disabilities and autistic people have due to their neurodevelopmental condition, comorbidity renders differential diagnosis and treatments more complex, particularly if unrecognised, hence it is essential that clinicians are aware of the additional conditions that their patients may have.

Strengths and limitations

Strengths of the e-cohorts include their large scale, national Census coverage of the whole country of Scotland, with 94% uptake, and a high level of successful record linkage (>92%), hence limiting bias, and the systematic enquiry about intellectual disabilities and autism of the whole population. The questions on intellectual disabilities and autism were subject to cognitive question testing prior to use, to ensure that they accurately captured the conditions, and were acceptable to the population. Additionally, specific learning disabilities were distinguished from intellectual disabilities or autism. Scotland has a rich profile of routinely collected health data which has been successfully used many times in previous research on the general population, and linkage of Scotland's Census, 2011 to these data, has provided large scale, whole country cohorts of intellectual disabilities, and of autism. Limitations include that routinely collected data lack disease-specificity and granularity, and cause of death data is infrequently verified by post-mortem. Ascertainment of intellectual disabilities and autism was based on self or proxy reporting. Scotland is a high income country with good country-wide educational and diagnostic services, and diagnosis of neurodevelopmental conditions attracts additional support for learning so is advantageous for the child. If a child's learning is delayed, schools have a statutory responsibility to seek assessment, so such children will have received a formal diagnosis. Proxy-reporting is the basis for much of the

For peer review only - http://bmjopen.bmj.com/site/about/guidelines.xhtml

healthcare provided for people with intellectual disabilities and autistic people who cannot self-report, and overall, proxy reports are concluded to be a useful addition to determine aspects of wellbeing when the need arises.[40] Those adults for whom a diagnosis of autism was recorded will reflect the contemporary diagnostic practices in place during their childhood; and the concept of the autism spectrum has broadened in recent years.

Collaboration

The breadth of the datasets created means they are suitable to be repurposed to answer many additional questions to the ones we outline in our current programme of research. Collaboration is encouraged, and as we are not the data controllers, this would be subject to approval by Scotland's Public Benefit and Privacy Panel for Health, Scotland's Statistics Public Benefit and Privacy Panel, and ethical approval by an appropriate ethical committee.

Ethics statement

This programme of research was approved by Scotland's Public Benefit and Privacy Panel for Health (reference: 1819-0051), Scotland's Statistics Public Benefit and Privacy Panel (1819-0051), and the University of Glasgow's College of Medical, Veterinary, and Life Sciences Ethical Committee (reference: 200180081). Data sharing agreements are in place with the data controllers of all the linked datasets.

Contributorship statement

S-AC conceived and designed the programme, and wrote the first draft of the manuscript. AH and DK contributed to the design, and project management. DMcK, MF, and GS designed and supervised the statistics. LH-McC, ER, and KD analysed data. JP contributed expertise on data linkage and datasets. CM oversees programme management. All contributed to drafting the manuscript, and approved the final version. S-AC is the guarantor.

Competing interests

The authors declare no competing interests.

Funding

This work was supported by the UK Medical Research Council, grant number: MC_PC_17217, the Scottish Government via the Scottish Learning Disabilities Observatory, and the Baily Thomas charitable fund.

The study sponsor and funders had no role in the study design; in the collection, analysis, and interpretation of data; in the writing of the report; and in the decision to submit the article for publication.

The researchers are independent from the funders.

Data sharing statement

For peer review only

Data access is available through approval by Scotland's Public Benefit and Privacy Panel for Health, Scotland's Statistics Public Benefit and Privacy Panel, and an appropriate ethical committee.

Acknowledgements

The authors would like to acknowledge the support of the eDRIS Team (Public Health Scotland), in particular David Clark, for their involvement in obtaining approvals, provisioning and linking data, and for the use of the secure analytical platform within the National Safe Haven.

Word count

2,902

References

1. O'Leary L, Hughes-McCormack L, Cooper S-A. Early death and causes of death of people with intellectual disabilities: A systematic review. *J Appl Res Intellect Disabil* 2017;31:325-42.
2. O'Leary L, Hughes-McCormack L, Cooper S-A. Life expectancy and causes of death of people with Down syndrome: a systematic review. *J Appl Res Intellect Disabil* 2018;31:687-708.
3. Glover G, Williams R, Heslop P, *et al.* Mortality in people with intellectual disabilities in England. *J Intellect Disabil Res* 2017;61:62-74.
4. Cooper S-A, Allan L, Greenlaw, N, *et al.* Rates, causes, place, and predictors of death in adults with intellectual disabilities with and without Down syndrome: prospective cohort study with record linkage. *BMJ Open* 2020;10:e036465.
5. Smith GS, Fleming M, Kinnear D, *et al.* Rates and causes of mortality among children and young people with and without intellectual disabilities in Scotland: a record linkage cohort study of 796,190 schoolchildren. *BMJ Open* 2020;P10e034077:doi: 10.1136/bmjopen-2019-034077
6. Mouridsen SE, Brønnum-Hansen H, Rich B, Isager T. Mortality and causes of death in autism spectrum disorders: an update. *Autism* 2008;12:403-14.
7. Hirvikoski T, Mittendorfer-Rutz E, Boman M, Larsson H, Lichtenstein P, Bölte S. Premature mortality in autism spectrum disorder. *Br J Psychiatry* 2016;208:232-238.
8. Hwang YI, Srasuebkul P, Foley KR, Arnold S, Troller JN. Mortality and cause of death of Australians on the autism spectrum. *Autism Res* 2019;12:9806-815.
9. Smith GS, Fleming M, Kinnear D, *et al.* Mortality in 787,666 school pupils in Scotland with and without autism: a cohort study. *Autism* 2021;25:300–304.
10. Heslop P, Blair PS, Fleming P, *et al.* The Confidential Inquiry into premature deaths of people with intellectual disabilities in the UK: a population-based study. *Lancet* 2014;383:889–95.
11. Hosking FJ, Carey IM, Shah SM, *et al.* Mortality among adults with intellectual disability in England: Comparisons with the general population. *Am J Public Healt,* 2016;106:1483–90. ERRATUM: *Am J Public Health* 2016;106:e11–e12. doi: 10.2105/AJPH.2016.303240e
12. Cooper S-A, Hughes-McCormack L, Greenlaw N, *et al.* Management and prevalence of long-term conditions in primary health care for adults with intellectual disabilities compared with the general population: a population-based cohort study. *J Appl Res Intellect Disabil* 2017;31(S1):68–81. 10.1111/jar.12386

For peer review only - http://bmjopen.bmj.com/site/about/guidelines.xhtml

13. Kinnear D, Morrison J, Allan L, *et al.* The prevalence of multi-morbidity in a cohort of adults with intellectual disabilities, with and without Down syndrome. *BMJOpen* 2018; 0:e018292.
14. Cooper S-A, McLean G, Guthrie B, *et al.* Multiple physical and mental health comorbidity in adults with intellectual disabilities: population-based cross-sectional analysis. *BMC Family Practice* 2015;DOI:10.1186/s12875-015-0329-3
15. Lunsky Y, Lin E, Balogh R, Klein-Geltink J. Diabetes prevalence among persons with serious mental illness and developmental disability. *Psychiatric Service* 2011;62:830.
16. McVilly K, McGillivray J, Curtis A, Lehman J, Morrish L, Speight J. Diabetes in people with an intellectual disability: a systematic review of prevalence, incidence and impact. *Diabetic Med* 2014;31:897-904.
17. Tyrer F, Dunkley AJ, Singh J. *et al*. Multimorbidity and lifestyle factors among adults with learning disability: a cross-sectional analysis of a UK cohort. *J Intellect Disabil Res* 2019;63:255-265.
18. Melville CA, McGarty A, Harris L, *et al.* A population based study of the prevalence and correlates of sedentary behaviour of adults with intellectual disabilities. *J Intellect Disabil Res* 2018;62:60-71.
19. Sullivan SG, Hussain R, Threlfall T, Bittles AH. The incidence of cancer in people with intellectual disabilities. *Cancer Causes & Control* 2004;15:1021-1025.
20. Patja K, Eero P, Iivanainen M. Cancer incidence among people with intellectual disability. *J Intellect Disabil Res* 2001;45:300-7.
21. Böhmer CJM, Klinkenberg-Knol EC, Niezen-de-Boer RC, Meuwissen SG. The age-related incidence of oesophageal carcinoma in intellectually disabled individuals in institutions in the Netherlands. *European J Gastroenterology and Hepatology* 1997;9:589-92.
22. Hughes-McCormack LA, Rydzewska E, Henderson A, *et al.* Prevalence and general health status of people with intellectual disabilities in Scotland -a total population study. *J Epidemiology Community Health* 2017;72:78–85.
23. Rydzewska E, Hughes-McCormack LA, Gillberg C, *et al.* General health of adults with autism spectrum disorder -a whole country population cross-sectional study. *Res Autism Spectrum Disorders* 2019;60:59-66.
24. Rydzewska E, Hughes-McCormack LA, Gillberg C, *et al.* Age at identification, prevalence and general health of children with autism: observational study of a whole country population. *BMJOpen* 2019;9:e025904.
25. Rydzewska E, Dunn K, Cooper S-A. An umbrella systematic review on systematic reviews and meta-analyses on comorbid health conditions in people with autism spectrum disorder. *Br J Psychiatry* 2021;218:10-19.
26. Ali A, Scior K, Ratti V, Strydom A, King M, Hassiotis A. Discrimination and other barriers to accessing health care: perspectives of patients with mild and moderate intellectual disability and their carers. *PLoS ONE* 2013;8(8):e70855. https://doi.org/10.1371/journal.pone. 0070855
27. Calleja S, Islam FMA, Ingsley J, McDonald R. Healthcare access for autistic adults. A systematic review. *Med* 2020;99(29):e20899
28. Cobigo V, Oullette-Kuntz H, Balogh R, Leung F, Lin E, Lunsku Y. Are cervical and breast cancer screening programmes equitable? The case of women with intellectual disabilities and developmental disabilities. *J Intellect Disabil Res* 2013;57:478–488.
29. Osborn DPJ, Horsfall L, Hassiotis A, Petersen I, Walters K, Nazareth I. Access to cancer screening in people with learning disabilities in the UK: cohort study in the health improvement network, a primary care research database. *PLoS ONE* 2012;7(8):e43841. DOI: 10.1371/journal.pone.0043841

For peer review only - http://bmjopen.bmj.com/site/about/guidelines.xhtml

30. Dunn K, Hughes-McCormack L, Cooper S-A. Hospital admissions for physical health conditions for people with intellectual disabilities: Systematic Review. *J App Res Intellect Disabil* 2017;31 (S1):1–10. DOI: 10.1111/jar.12360
31. Hand BN, Boan AD, Bradley CC, Charle, JM, Carpenter LA. Ambulatory care sensitive admissions in individuals with autism spectrum disorder, intellectual disability, and population controls. *Autism Res* 2019;12:295-302.
32. National Records of Scotland (NRS). *2011 Census Release 1C - How the 2011 Census population estimates were obtained*. Edinburgh: National Records of Scotland 2013.
33. World Health Organisation. *International Statistical Classification of Diseases and Related Health Problems, 10th Revision.* Geneva: World Health Organisation, 1992.
34. Kinnear D, Rydzewska E, Dunn K, *et al.* The relative influence of intellectual disabilities and autism on mental and general health. A cross-sectional study of a whole country of 5.3 million children and adults. *BMJ Open* 2019;9(8):e029040.
35. Dunn KD, Rydzewska E, Rintoul J, Macintyre C, Cooper S-A. The prevalence and general health status of people with intellectual disabilities and autism co-occurring together: a total population study. *J Intellect Disabil Res* 2019;63:277-285.
36. Hughes-McCormack L, Rydzewska E, Henderson A, Rintoul J, Macintyre C, Cooper S-A. Prevalence of mental ill-health and relationship with physical health in a whole country population of 26,349 people with intellectual disabilities, compared with the general population. *Br J Psychiatry Open* 2017;3,Issue 5:http://bjpo.rcpsych.org/content/3/5/243
37. Rydzewska E, Hughes-McCormack LA, Gillberg C, *et al.* Prevalence of long-term health conditions in adults with autism - observational study of a whole country population. *BMJOpen* 2018;8:e023945.
38. Dunn K, Rydzewska E, Cooper S-A. The prevalence of mental health conditions, sensory impairments, and physical disability in people with co-occurring intellectual disabilities and autism compared with other people – a total population study of 5,295,403 people. *BMJ Open* 2020;10:e035280.
39. Rydzewska E, Hughes-McCormack LA, Gillberg C, *et al.* Prevalence of sensory impairments, physical and intellectual disabilities, and mental health in children and young people with self/proxy-reported autism - observational study of a whole country population. *Autism* 2019;23(5):1201-1209.
40. Perkins EA. Self- and proxy reports across three populations: older adults, persons with Alzheimer's disease, and persons with intellectual disabilities. *J Policy Practice Intellect Disabil* 2007;4:1-10.

For peer review only

For peer review only - http://bmjopen.bmj.com/site/about/guidelines.xhtml

Table 1. Linked data sources included in the two datasets

DATASET 1

Resource	Time period	Use	Data controller
Scotland's Census, 2011	27.3.11	To identify the populations with intellectual disabilities, and autism, and the comparison population, and their basic demography	National Records of Scotland
Register of Deaths	27.3.11-15.08.20	To identify deaths, causes of deaths, and amenable deaths	National Records of Scotland
Scottish Morbidity Records 06	27.3.11-15.08.20	To identify cancer incidence, mortality, and treatments (quality of health care)	NHS National Services Scotland, Information Services Division

DATASET 2

Resource	Time period	Use	Data controller
Scotland's Census, 2011	27.3.11	To identify the populations with intellectual disabilities, and autism, and the comparison population, their basic demography, household, employment, and long-term conditions data (potential determinants of mortality)	National Records of Scotland
Register of Deaths	27.3.11-15.08.20	To identify deaths	National Records of Scotland
Prescribing Information System	27.3.11-27.9.11	To identify people with: asthma/COPD; angina/congestive heart failure/hypertension; peptic ulcer/reflux; constipation; diabetes; thyroid disorder; depression; bipolar disorders; anxiety/sleep; psychosis; ADHD; epilepsy; glaucoma (health burden)	NHS National Services Scotland, Information Services Division
Scottish Morbidity Records 01	27.3.11-15.08.20	To identify general/acute hospital admissions and causes, and ambulatory care sensitive admissions	NHS National Services Scotland, Information Services Division
Scottish Morbidity Records 04	27.3.11-15.08.20	To identify mental health admissions and their causes	NHS National Services Scotland, Information Services Division
SCI-diabetes	27.3.11-15.08.20	To identify quality of diabetic care and diabetic outcomes (quality of health care)	Caldicott guardians
National Bowel Screening Programme	27.3.11-15.08.20	To identify screening uptake	NHS National Services Scotland, Information Services Division
Cervical screening	2016-15.08.20	To identify screening uptake	NHS National Services Scotland, Information Services Division

For peer review only - http://bmjopen.bmj.com/site/about/guidelines.xhtml

Table 2. Characteristics of the cohorts

Variable	Category	Dataset 1 and 2 Intellectual disabilities N=22,538 (100%)	Dataset 1 and 2 Autism N=27,741 (100%)	Dataset 1 15% of general population† N=700,437 (100%)	Dataset 2 5% of general population† N=233,378 (100%)
**Sex**	Male	12,837 (57.0%)	21,390 (77.1%)	335,226 (47.9%)	111,497 (47.8%)
	Female	9,701 (43.0%)	6,351 (22.9%)	365,211 (52.1%)	121,881 (52.2%)
**Age**	0-15	4,509 (20.0%)	15,387 (55.5%)	117,980 (16.8%)	39,220 (16.8%)
	15-24	3,546 (15.7%)	6,790 (24.5%)	76,184 (10.9%)	25,574 (11.0%)
	25-34	2,976 (13.2%)	2,024 (7.3%)	83,868 (12.0%)	27,721 (11.8%)
	35-45	3,277 (14.5%)	1,300 (4.7%)	97,886 (14.0%)	32,599 (14.0%)
	45-54	3,664 (16.3%)	1,053 (3.8%)	108,052 (15.4%)	36,164 (15.5%)
	55-64	2,494 (11.1%)	617 (2.2%)	92,698 (13.2%)	31,022 (13.3%)
	65-74	1,330 (5.9%)	300 (1.1%)	67,532 (9.6%)	22,166 (9.5%)
	75+	742 (3.3%)	270 (1.0%)	56,237 (8.0%)	18,912 (8.1%)
**Ethnicity***	White	22,022 (97.7%)	26,976 (97.2%)	Not available	225,626 (96.7%)
	Asian/Asian Scottish/Asian British	368 (1.6%)	425 (1.5%)	Not available	5,181 (2.2%)
	African	35 (0.2%)	80 (0.3%)	Not available	928 (0.4%)
	Caribbean or black	21 (0.1%)	24 (0.1%)	Not available	213 (0.1%)
	Mixed/multiple ethnic groups	66 (0.3%)	189 (0.7%)	Not available	888 (0.4%)
	Other ethnic group	26 (0.1%)	47 (0.2%)	Not available	542 (0.2%)
**SIMD**	1 – most deprived	6,200 (27.5%)	6,748 (24.3%)	130,354 (18.6%)	42,828 (18.4%)
	2	5,481 (24.3%)	5,901 (21.3%)	135,311 (19.3%)	45,407 (19.5%)
	3	4,516 (20.0%)	5,678 (20.5%)	142,238 (20.3%)	47,886 (20.5%)
	4	3,693 (16.4%)	5,128 (18.5%)	147,866 (21.1%)	49,429 (21.2%)
	5 – most affluent	2,648 (11.7%)	4,286 (15.5%)	144,668 (20.7%)	47,828 (20.5%)
**General health status***	Very good	3,389 (15.0%)	10,510 (38.0%)	Not available	122,511 (52.4%)
	Good	7,942 (35.2%)	9,608 (34.6%)	Not available	69,575 (30.0%)
	Fair	7,715 (34.2%)	5,387 (19.4%)	Not available	28,273 (12.1%)
	Bad	2,356 (10.5%)	1,533 (5.5%)	Not available	9,885 (4.2%)
	Very bad	1,136 (5.0%)	703 (2.5%)	Not available	3,134 (1.3%)
**Mental health condition***	Yes	4,755 (21.1%)	3,998 (14.4%)	Not available	9,710 (4.2%)
	No	17,783 (78.9%)	23,743 (85.6%)	Not available	223,668 (95.8%)

SIMD=Scottish Index of Multiple Deprivation
†General population with neither intellectual disabilities nor autism
*Data available for Dataset 2 only

For peer review only - http://bmjopen.bmj.com/site/about/guidelines.xhtml

Figure. Example power calculations across comparison sample sizes

For peer review only

For peer review only - http://bmjopen.bmj.com/site/about/guidelines.xhtml

Females only

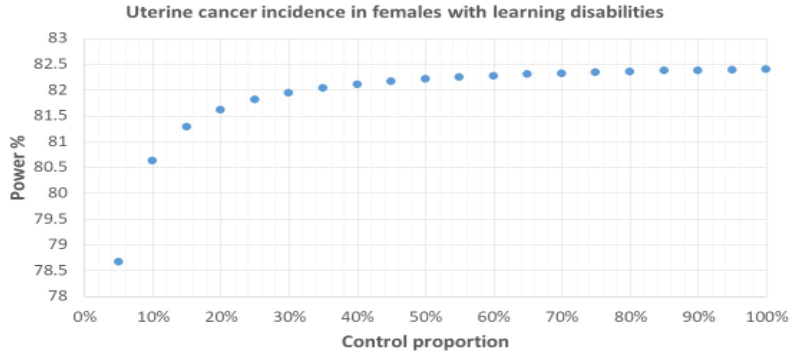

- Proportion of control population - 5% (n=135,838) to 100% n=(2,716,759)
- Based on uterine cancer incidence rate of 29.1 cases per 100,000 in the female general population
- Standardised mortality ratio of 2.98 (Sullivan et al. 2004) in 11,200 females with learning disabilities

Males only

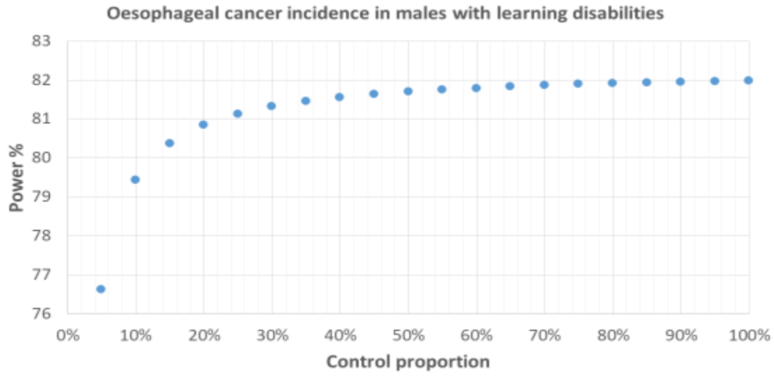

- Proportion of control population –5% (n=127,615) to 100% (n=2,552,295)
- Based on oesophageal cancer incidence rate of 22.8 cases per 100,000 in general population males
- Standardised mortality ratio of 2.9 (Böhmer et al. 1997) in 15,149 males with learning disabilities

Males only

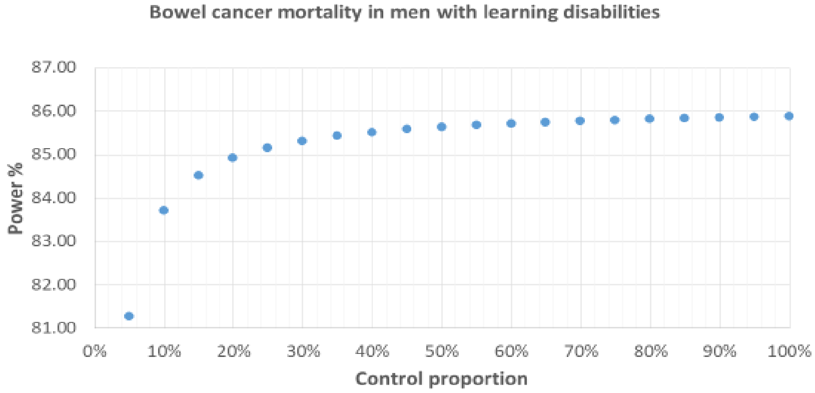

- Proportion of control population –5% (n=127,615) to 100% (n=2,552,295)
- Based on annual Scottish cancer mortality rate of 32.6 deaths per 100,000
- Standardised mortality ratio of 2.68 (Glover et al. 2015) in 15,149 males with learning disabilities

Supplementary file 1. List of variables included in the two datasets

DATASET 1

Type of variable	Outcome	Exposure	Potential confounding factors
**Scotland's Census 2011**			
Read-through Index number for linkage		N/A	N/A
Month and year of birth			X
Living arrangement - seven exclusive values			X
Scottish Index of Multiple Deprivation 2012			X
Urban-rural classification 8-fold 2011-12			X
Heath Board area 2011			X
Council area 2011			X
Long-term health condition: autism		X	
Long-term health condition: learning disability		X	
Sex			X
**NRS Death register**			
CHI		N/A	N/A
Age at death	X		
Main cause of death	X		
All causes of death (1-11)	X		
Date of death	X		
Scottish Index of Multiple Deprivation 2012			X
Place of death			X
**SMR06**			
CHI		N/A	N/A
Date of incidence	X		
Date of registration	X		
Death certificate only	X		
Side			X
Site (ICD09/ICD10/ICD02)	X		
Type ICD0/ICD02/ICD03	X		
Treated with surgery			X
Treated with radiotherapy			X
Treated with chemotherapy			X
Treated with hormones			X
Treated with other therapy			X

For peer review only

DATASET 2

Type of variable	Outcome	Exposure	Potential confounding factors
**Scotland's Census 2011**			
Read-through Index number for Census 2011		N/A	N/A
Month and year of birth			X
Living arrangements - seven exclusive values			X
Scottish Index of Multiple Deprivation 2012			X
Urban-rural classification 8-fold 2011-12			X
Heath Board area 2011			X
Council area 2011			X
Activity last week			X
Adults in employment, number in household			X
Adults in household, number of			X
Carers in household with economic activity			X
Provision of unpaid care			X
Cars or vans, number of			X
Central heating type in household			X
Unpaid carers in household, number of			X
Limitation of activities			X
Distance travelled to work or place of study			X
Recorded ethnicity			X
Nature of communal establishment			X
Ever employed			X
Number of residents in family			X
Family status			X
Count of dependent children in family			X
General Health status			X
Highest level of qualification			X
Hours worked (grouped)			X
Language proficiency			X
Marital status			X
Long-term health condition: blind		X	X
Long-term health condition: autism		X	X
Long-term health condition: deaf		X	X
Long-term health condition: dyslexia		X	X
Long-term health condition: learning disability		X	X
Long-term health condition: mental health condition		X	X
Long-term health condition: physical disability		X	X

For peer review only

Type of variable	Outcome	Exposure	Potential confounding factors
Occupancy rating			X
Occupancy status			X
Parent to household member			X
Population density of datazone			X
Persons per room, number in household			X
Sex			X
Household size			X
Students living away from home during term time (aged 4 and over)			X
Schoolchild or full-time student indicator			X
Tenure of household			X
Term-time address indicator			X
Accommodation type			X
**NRS Death register**			
CHI		N/A	N/A
Age at death	X		
Main cause of death	X		
All causes of death (1-11)	X		
Month of death	X		
Scottish Index of Multiple Deprivation 2012			X
Place of death			X
**Prescribing Information System**			
CHI		N/A	N/A
Date medication prescribed			X
Date medication dispensed			X
Date medication paid			X
BNF codes of drugs for: asthma/COPD; angina/congestive heart failure/hypertension; peptic ulcer/reflux; constipation; diabetes; thyroid dysfunction; depression; bipolar disorder; anxiety/sleep; psychosis; ADHD; epilepsy; glaucoma		X	X
BNF root drug		X	
Prescribed item name/approved name		X	
Prescribed item strength			X
Number of defined daily doses dispensed			X
Number of dispensed/paid items			X
Dispensed/paid quantity			X
**SMR01**			
CHI		N/A	N/A
Date of admission	X		
Date of discharge	X		
Main condition on discharge	X		

For peer review only

Type of variable	Outcome	Exposure	Potential confounding factors
Secondary condition on discharge (1-5)	X		
Main operation	X		
Other operations (1-3)	X		
Length of stay	X		
CIS marker	X		
Inpatient/day case marker	X		
Type of facility/management patient			X
Type of admission	X		
Admission reason	X		
**SMR04**			
CHI		N/A	N/A
Date of admission	X		
Date of discharge	X		
Main condition on discharge	X		
Secondary condition on discharge (1-5)	X		
Main operation	X		
Other operations (1-3)	X		
Main condition on admission	X		
Other conditions on admission (1-3)	X		
Previous psychiatric care			X
Type of psychiatric care			X
**SCI-Diabetes**			
CHI		N/A	N/A
Date of diagnosis			X
Type of diabetes		X	
HbA1c	X		
Hypoglycaemic coma	X		
Attendance at hospital diabetes clinic	X		
Arrangements for formal diabetes care	X		
Drugs			X
Date of death	X		
Cause of death	X		
Underlying cause of death	X		
Weight			X
Height			X
Body Mass Index			X
Systolic bp			X
Diastolic bp			X
Smoking status at date of contact			X
Year patient stopped smoking			X
Alcohol intake			X
Diabetic review on this date	X		
Referral to defined health care professional	X		
Seen by defined health care professional	X		

For peer review only

Type of variable	Outcome	Exposure	Potential confounding factors
**National Bowel Screening programme**			
CHI		N/A	N/A
Health board of residence			X
Date screening test kit sent			X
Screening test result	X		
Colonoscopy performed?	X		
Reason for not having a colonoscopy	X		
Colonoscopy completed	X		
Invasive cancer detected?	X		
ICD 10 classification of neoplasm	X		
Complication arising from colonoscopy			X
TNM derived Dukes' stage	X		
Polyps detected	X		
Adenoma detected	X		
**Cervical Cancer screening**			
CHI		N/A	N/A
Date of test result	X		
SIMD 2012 deprivation category			X
Date of HPV Immunisation	X		
HPV Immunisation Status	X		
Screened within 3.5yr	X		
Screened within 5.5yr	X		

For peer review only

Supplementary file 2. Data processing flowchart

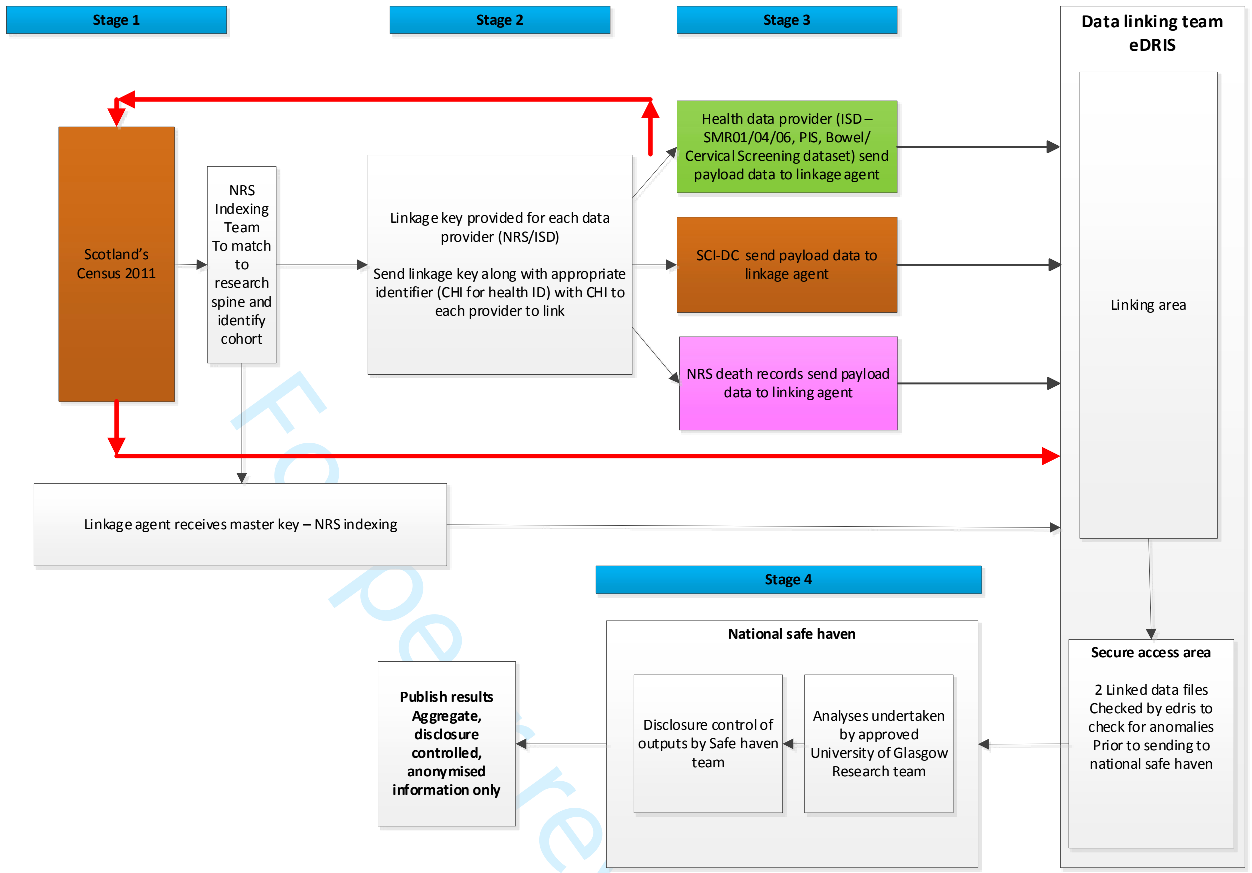

CHI=community health index; eDRIS=electronic data research and innovation service; ISD=information services division of national services Scotland; NRS=national records of Scotland; PIS=prescribing information system; SCI-DC=Scottish care information–diabetes collaboration; SMR=Scottish morbidity record

For peer review only

STROBE (Strengthening The Reporting of OBservational Studies in Epidemiology) Checklist

A checklist of items that should be included in reports of observational studies. You must report the page number in your manuscript where you consider each of the items listed in this checklist. If you have not included this information, either revise your manuscript accordingly before submitting or note N/A.

Note: An Explanation and Elaboration article discusses each checklist item and gives methodological background and published examples of transparent reporting. The STROBE checklist is best used in conjunction with this article (freely available on the Web sites of PLoS Medicine at http://www.plosmedicine.org/, Annals of Internal Medicine at http://www.annals.org/, and Epidemiology at http://www.epidem.com/). Information on the STROBE Initiative is available at www.strobe-statement.org.

Section and Item	Item No.	Recommendation	Reported on Page No.
**Title and Abstract**	1	(*a*) Indicate the study's design with a commonly used term in the title or the abstract	
		(*b*) Provide in the abstract an informative and balanced summary of what was done and what was found	
**Introduction**			
Background/Rationale	2	Explain the scientific background and rationale for the investigation being reported	
Objectives	3	State specific objectives, including any prespecified hypotheses	
**Methods**			
Study Design	4	Present key elements of study design early in the paper	
Setting	5	Describe the setting, locations, and relevant dates, including periods of recruitment, exposure, follow-up, and data collection	
Participants	6	(*a*) *Cohort study*—Give the eligibility criteria, and the sources and methods of selection of participants. Describe methods of follow-up<br>*Case-control study*—Give the eligibility criteria, and the sources and methods of case ascertainment and control selection. Give the rationale for the choice of cases and controls<br>*Cross-sectional study*—Give the eligibility criteria, and the sources and methods of selection of participants	
		(*b*) *Cohort study*—For matched studies, give matching criteria and number of exposed and unexposed<br>*Case-control study*—For matched studies, give matching criteria and the number of controls per case	
Variables	7	Clearly define all outcomes, exposures, predictors, potential confounders, and effect modifiers. Give diagnostic criteria, if applicable	

For peer review only

Section and Item	Item No.	Recommendation	Reported on Page No.
Data Sources/ Measurement	8*	For each variable of interest, give sources of data and details of methods of assessment (measurement). Describe comparability of assessment methods if there is more than one group	
Bias	9	Describe any efforts to address potential sources of bias	
Study Size	10	Explain how the study size was arrived at	
Quantitative Variables	11	Explain how quantitative variables were handled in the analyses. If applicable, describe which groupings were chosen and why	
Statistical Methods	12	(*a*) Describe all statistical methods, including those used to control for confounding	
		(*b*) Describe any methods used to examine subgroups and interactions	
		(*c*) Explain how missing data were addressed	
		(*d*) *Cohort study*—If applicable, explain how loss to follow-up was addressed<br>*Case-control study*—If applicable, explain how matching of cases and controls was addressed<br>*Cross-sectional study*—If applicable, describe analytical methods taking account of sampling strategy	
		(*e*) Describe any sensitivity analyses	
**Results**			
Participants	13*	(a) Report numbers of individuals at each stage of study—eg numbers potentially eligible, examined for eligibility, confirmed eligible, included in the study, completing follow-up, and analysed	
		(b) Give reasons for non-participation at each stage	
		(c) Consider use of a flow diagram	
Descriptive Data	14*	(a) Give characteristics of study participants (eg demographic, clinical, social) and information on exposures and potential confounders	
		(b) Indicate number of participants with missing data for each variable of interest	
		(c) *Cohort study*—Summarise follow-up time (eg, average and total amount)	
Outcome Data	15*	*Cohort study*—Report numbers of outcome events or summary measures over time	
		Case-control study—Report numbers in each exposure category, or summary measures of exposure	
		Cross-sectional study—Report numbers of outcome events or summary measures	

For peer review only

Section and Item	Item No.	Recommendation	Reported on Page No.
Main Results	16	(*a*) Give unadjusted estimates and, if applicable, confounder-adjusted estimates and their precision (eg, 95% confidence interval). Make clear which confounders were adjusted for and why they were included	
		(*b*) Report category boundaries when continuous variables were categorized	
		(*c*) If relevant, consider translating estimates of relative risk into absolute risk for a meaningful time period	
Other Analyses	17	Report other analyses done—eg analyses of subgroups and interactions, and sensitivity analyses	
**Discussion**			
Key Results	18	Summarise key results with reference to study objectives	
Limitations	19	Discuss limitations of the study, taking into account sources of potential bias or imprecision. Discuss both direction and magnitude of any potential bias	
Interpretation	20	Give a cautious overall interpretation of results considering objectives, limitations, multiplicity of analyses, results from similar studies, and other relevant evidence	
Generalisability	21	Discuss the generalisability (external validity) of the study results	
**Other Information**			
Funding	22	Give the source of funding and the role of the funders for the present study and, if applicable, for the original study on which the present article is based	

*Give information separately for cases and controls in case-control studies and, if applicable, for exposed and unexposed groups in cohort and cross-sectional studies.

Once you have completed this checklist, please save a copy and upload it as part of your submission. DO NOT include this checklist as part of the main manuscript document. It must be uploaded as a separate file.

For peer review only

BMJ Open

Cohort profile: Scotland’s record-linkage e-cohorts of people with intellectual disabilities, and autistic people (SCIDA)

Journal:	*BMJ Open*
Manuscript ID	bmjopen-2021-057230.R1
Article Type:	Cohort profile
Date Submitted by the Author:	27-Jan-2022
Complete List of Authors:	Cooper, Sally-Ann; University of Glasgow College of Medical Veterinary and Life Sciences, Institute of Health and Wellbeing<br>Henderson, Angela; University of Glasgow, Institute of Health and Wellbeing<br>Kinnear, Deborah; University of Glasgow, Institute of Health and Wellbeing<br>Mackay, Daniel; University of Glasgow,<br>Fleming, Michael ; University of Glasgow, Public Health<br>Smith, Gillian; University of Glasgow Institute of Health and Wellbeing, Mental Health and Wellbeing<br>Hughes-McCormack, Laura; University of Glasgow, Institute of Health and Wellbeing<br>Rydzewska, Ewelina; University of Glasgow, Institute of Health and Wellbeing<br>Dunn, Kirsty; University of Glasgow, Institute of Health and Wellbeing<br>Pell, J. P.; Univ Glasgow<br>Melville, Craig; University of Glasgow
**Primary Subject Heading**:	Research methods
Secondary Subject Heading:	Epidemiology
Keywords:	Quality in health care < HEALTH SERVICES ADMINISTRATION & MANAGEMENT, MENTAL HEALTH, PUBLIC HEALTH

SCHOLARONE™
Manuscripts

I, the Submitting Author has the right to grant and does grant on behalf of all authors of the Work (as defined in the below author licence), an exclusive licence and/or a non-exclusive licence for contributions from authors who are: i) UK Crown employees; ii) where BMJ has agreed a CC-BY licence shall apply, and/or iii) in accordance with the terms applicable for US Federal Government officers or employees acting as part of their official duties; on a worldwide, perpetual, irrevocable, royalty-free basis to BMJ Publishing Group Ltd ("BMJ") its licensees and where the relevant Journal is co-owned by BMJ to the co-owners of the Journal, to publish the Work in this journal and any other BMJ products and to exploit all rights, as set out in our licence.

The Submitting Author accepts and understands that any supply made under these terms is made by BMJ to the Submitting Author unless you are acting as an employee on behalf of your employer or a postgraduate student of an affiliated institution which is paying any applicable article publishing charge ("APC") for Open Access articles. Where the Submitting Author wishes to make the Work available on an Open Access basis (and intends to pay the relevant APC), the terms of reuse of such Open Access shall be governed by a Creative Commons licence – details of these licences and which Creative Commons licence will apply to this Work are set out in our licence referred to above.

Other than as permitted in any relevant BMJ Author's Self Archiving Policies, I confirm this Work has not been accepted for publication elsewhere, is not being considered for publication elsewhere and does not duplicate material already published. I confirm all authors consent to publication of this Work and authorise the granting of this licence.

For peer review only

Cohort profile: Scotland's record-linkage e-cohorts of people with intellectual disabilities, and autistic people (SCIDA)

***Sally-Ann Cooper**
Angela Henderson
Deborah Kinnear
Danny MacKay
Michael Fleming
Gillian Smith
Laura Hughes-McCormack
Ewelina Rydzewska
Kirsty Dunn
Jill P Pell
Craig Melville

*Corresponding author:
Sally-Ann Cooper
Institute of Health and Wellbeing, University of Glasgow,
1st Floor Administrative Building, Gartnavel Royal Hospital, 1,055 Great Western Road, Glasgow, UK
Sally-Ann.Cooper@glasgow.ac.uk

For peer review only

Abstract

Purpose
To investigate health, mortality, and health-care inequalities experienced by people with intellectual disabilities, and autistic people, and their determinants; an important step towards identifying and implementing solutions to reduce inequalities. This paper describes the cohorts, record-linkages, and variables that will be used.

Participants
Scotland's Census, 2011 was used to identify Scotland's citizens with intellectual disabilities, and autistic citizens, and representative general population samples with neither. Using Scotland's community health index, the Census data (demography, household, employment, long-term conditions) were linked with routinely collected health, death, and health-care data: Scotland's register of deaths, Scottish morbidity data 06 (SMR06: cancer incidence, mortality, treatments), Prescribing Information System (identifying asthma/COPD; angina/congestive heart failure/hypertension; peptic ulcer/reflux; constipation; diabetes; thyroid disorder; depression; bipolar disorders; anxiety/sleep; psychosis; attention deficit hyperactivity disorder; epilepsy; glaucoma), SMR01 (general/acute hospital admissions and causes, ambulatory care sensitive admissions), SMR04 (mental health admissions and causes), Scottish Care Information–Diabetes Collaboration (diabetic care quality, diabetic outcomes), national bowel screening programme, and cervical screening.

Findings to date
Of the whole population, 0.5% had intellectual disabilities, and 0.6% were autistic. Linkage was successful for >92%. The resultant e-cohorts include: (1) 22,538 people with intellectual disabilities (12,837 male, 9,701 female), 4,509 of whom are children <16 years, (2) 27,741 autistic people (21,390 male, 6,351 female), 15,387 of whom are children <16 years, and (3) representative general population samples with neither condition. Very good general health was reported for only 3,389 (15.0%) people with intellectual disabilities, 10,510 (38.0%) autistic people, compared with 52.4% general population. Mental health conditions were reported for 4,755 (21.1%) people with intellectual disabilities, 3,998 (14.4%) autistic people, compared with 4.2% general population.

Future plans
Analyses will determine the extent of premature mortality, causes of death, and avoidable deaths, profile of health conditions and cancers, health-care quality and screening, and determinants of mortality and health-care.

Strengths and limitations of this study

- 94% Census completion for a whole country population, and >92% successful record linkage.
- Record linkage across multiple data sources.
- Limitations include the use of routinely collected data.
- Reliance upon recorded cause of death on death certificates.
- Self/proxy reporting of intellectual disabilities and autism, and other Census data.

For peer review only

For peer review only - http://bmjopen.bmj.com/site/about/guidelines.xhtml

Introduction

People with intellectual disabilities are thought to die about 20 years earlier than other people,[1] or 28 years earlier for people with Down syndrome,[2] but most studies are small. A large English study found a standardised mortality ratio (SMR) of 3.18 (95% confidence intervals 2.94, 3.43).[3] Two large Scottish studies reported an SMR of 2.24 (1.98, 2.49) in adults overall; 5.28 (3.98, 6.57) for Down syndrome adults, and 1.93 (1.68, 2.18) for adults without Down syndrome,[4] and an SMR of 11.6 (9.6, 14.0) in children overall.[5] Autism is also thought to be associated with premature death, though there have been few studies, and co-occurring intellectual disabilities may account for some of the reported findings.[6-9] The commonest causes of death of people with intellectual disabilities are likely to differ from other people. For example, aspiration pneumonia which is avoidable, may occur quite commonly,[3,4,10] and SMRs have been reported as 2.4 (1.3, 3.8) for colorectal cancer, and 2.3 (1.0, 4.3) for female genital cancers[3]. Additionally, probably about 40% of early mortality would have been treatable by good quality health care, which may be double the amount that occurs in other people.[4,10,11] Causes of death of autistic people may also differ from those in the general population, with deaths related to the nervous system, and injury and poisoning reported to be the most common causes.[8] These inequalities are a major injustice that need addressing. An important step towards this is to gain a better understanding of health, mortality, health-care, and their determinants in people with intellectual disabilities and autistic people.

The health profiles of people with intellectual disabilities and autistic people differ from other people, which has a bearing on mortality. People with intellectual disabilities have, for example, high rates of epilepsy, mental illness, gastrointestinal disease, antipsychotic use, and polypharmacy.[12-14] They may have higher diabetes rates,[15,16] due to sedentary lifestyles, obesity, and antipsychotics.[17,18] Cancer profiles appear to differ from other people, with colorectal, stomach, oesophageal, brain, and uterine cancer, and leukaemia all having been reported to have higher incidence in people with intellectual disabilities, but with some inconsistencies between studies, and wide confidence intervals due to study sizes.[19-21] Higher incidence of gastrointestinal cancers is plausible, due to poor diets, lifestyle, and high rates of helicobacter pylori and gastro-oesophageal reflux disorder, and lower screening uptake. Multi-morbidity is typical in people with intellectual disabilities,[13,14] and poor health is reported for people with intellectual disabilities across the full life-course.[22] In autistic people, general health is also reported to be poor across the lifespan.[23,24] Autistic people commonly have co-morbid physical conditions, and some are more prevalent than in the general population, including sleep problems, epilepsy, sensory impairments, atopy, autoimmune disorders, and obesity, but not asthma.[25] However, there are substantial gaps in the evidence-base on the health of autistic people, and some findings are inconsistent.[25]

Despite this higher burden of health need and early mortality, health-care has been reported to be poorer for people with intellectual disabilities,[26] and for autistic people,[27] and barriers in access to health-care have been reported.[26,27] Adults with intellectual disabilities are reported to participate less in cervical screening and breast sceeening,[28] and bowel screening[29] than other people. There is also some indication that people with intellectual disabilities and autistic people are more likely to be hospitalised for ambulatory care-sensitive conditions than other people; these are conditions that should be fully manageable in primary health care settings, and so such hospitalisations are a further marker for poor care.[30,31] These factors may impact on poorer outcomes and survival, due to later stage disease when treatment is initiated, and poorer management of disease.

For peer review only - http://bmjopen.bmj.com/site/about/guidelines.xhtml

Internationally, one of the challenges in investigating these issues at a population level is the lack of data on representative samples of people with intellectual disabilities, or autistic people. Important studies have used novel methods to link routinely collected administrative data and identify people with intellectual disabilities via their use of educational services, services designed for adults with intellectual disabilities and/or though identifying diagnostic codes in health records likely to indicate the presence of intellectual disabilities or autism.[32-35] These approaches provide valuable data and also challenges, as, after leaving education/school, not all adults with intellectual disabilities use intellectual disabilities services, and nor do autistic adults, and there are coding issues in health records which are completed by multiple professionals and may be both incomplete and overinclusive for syndromes that are associated with intellectual disabilities in some but not all people. Additionally, caseness may be variably defined, with the term intellectual and developmental disorders being commonly used and which may or may not be a broader definition of neurodevelopmental disorders combining for example some or all of intellectual disabilities, autism, attention deficit hyperactivity disorder, and specific learning disabilities.[35] A further approach to population studies is using national surveys conducted with samples of the population, but has the limitation that people with intellectual disabilities or autism are represented in only small numbers and so restricts the analyses that can be undertaken, and in longitudinal research they tend to have higher cohort attrition and more biased cohort retention than other people.[35,36] In Scotland, the national Census in 2011 included questions on both intellectual disabilities and on autism, and so presents an ideal data-source for research with these populations. We are not aware of any other national Census data which has included data on intellectual disabilities and autism, other than Irish data which has the limitation of not distinguishing intellectual disabilities from specific learning disabilities.Record-linkage of Scotland's Census, 2011 to Scotland's other rich routinely collected health datasets and national statistics provides an excellent vehicle for detailed investigation of these issues. Scotland's Census, 2011 systematically questioned whether every person in the country had intellectual disabilities, and/or autism, and distinguished intellectual disabilities from dyslexia and other specific learning disabilities. It included data on 94% of the whole Scottish population, and imputed the remaining 6% (5,295,403 people in total). Due to its large size, it has power, when linked to other databases to answer important questions on health, causes of mortality, determinants, and health-care in people with intellectual disabilities, and autistic people. This is an important step leading towards a reduction in morbidity, early mortality and avoidable deaths, and improvements in health-care of people with intellectual disabilities and autistic people.

In Scotland's Census, 2011, of the whole Scottish population, 0.5% were recorded as having intellectual disabilities (0.4% females and 0.6% males), and 0.6% were recorded as being autistic people (0.3% female and 1.0% male). Regarding autism, 1.6% of all Scottish children and young people (<25 years of age) were recorded to have autism (0.7% females and 2.5% males), whilst 0.2% of adults aged 25 years and over were recorded to have autism (0.1% females and 0.3% males), reflecting the diagnostic broadening of autism spectrum disorders in recent years, and greater awareness of the condition. This also accounts for why, of the autistic children and young people, 15.0% additionally had intellectual disabilities, whilst of the autistic adults aged 25 years and over, 29.4% additionally had intellectual disabilities. In total, 1.1/1,000 people had both autism and intellectual disabilities, and are represented in both the intellectual disabilities and the autism cohorts. There is some evidence to suggest that autism is less recognised in females than in males,[37] but less so for people with more severe intellectual disabilities.[38]

For peer review only - http://bmjopen.bmj.com/site/about/guidelines.xhtml

We created two datasets, with record linkage to several of Scotland's health datasets. Linkage was undertaken for the 94% of the Scottish population who completed the Scottish Census, 2011. Of the people with intellectual disabilities recorded in Scotland's Census, 2011, 22,538 (92.9%) were successfully linked with their health records. Of the people with autism recorded in Scotland's Census, 2011, 27,741 (94.6%) were successfully linked with their health records. Regarding the general population who had neither intellectual disabilities nor autism, of the 15% randomly selected, 700,437 (95.1%) were successfully linked to their health records; whilst of the 5% randomly selected, 233,378 (95.1%) were successfully linked to their health records. Hence, each dataset includes data on 22,538 people with intellectual disabilities, 27,741 autistic people, and a randomly selected comparison general population with neither intellectual disabilities nor autism. For ethical reasons, two datasets were necessary to ensure data minimisation, as our research questions on cancer incidence and mortality required a larger comparison group to reach adequate power, due to lower incidence, than was required to answer the other research questions. The minimal number of variables was requested for each dataset: in dataset 1, this therefore did not include ethnicity, nor general or mental health variables, as there is not sufficient power to investigate these factors in relation to causes of deaths and cancers.

- Dataset 1 includes 22,538 people with intellectual disabilities, 27,741 autistic people, and a random sample of 700,437 (15%) of the Scottish population. It will be used to answer research questions on SMRs, causes of deaths, treatable deaths, cancer standardised incidence ratios (SIRs), and cancer SMRs.
- Dataset 2 includes 22,538 people with intellectual disabilities, 27,741 autistic people, and a random sample of 233,378 (5%) of the Scottish population. It will be used to answer research questions on independent predictors of deaths and treatable deaths, comparison of uptake of cervical screening and bowel screening, descriptions of population health burden, and quality of health-care.

The sample size for the comparison sample in dataset 1 was determined using the Scottish crude annual incidence rates and mortality rates for bowel cancer, oesophageal cancer, and uterine cancer as examples, and previously published SIRs and SMRs for people with intellectual disabilities[3,19,21]. The figure shows examples of some of the power graphs produced which led to the decision to use 15% of the Scottish population as the comparison group.

- Insert the figure about here -

The datasets we linked together and variables we selected were based on these specific research questions posed on (a) people with intellectual disabilities, and (b) autistic people, compared with other people:

1. What are the age and sex-standardised mortality ratios?
2. What are the common causes of death, and what proportion are treatable deaths?
3. Do cancer incidence and mortality differ?
4. What personal (including diseases identified via medication and admissions), household, and service-related factors (including hospital admissions) are markers/predictors of deaths and treatable deaths?
5. Do uptake of the national bowel and cervical cancer screening programmes differ?
6. What is the population health burden (identified via medication and admissions)?
7. Do people with diabetes* or other ambulatory care-sensitive conditions, receive as good health-care, and are diabetes or other ambulatory care-sensitive conditions more likely to result in hospital admissions and deaths?

*Diabetes was selected as an exemplar due to Scotland's Register of Diabetes Care.

For peer review only - http://bmjopen.bmj.com/site/about/guidelines.xhtml

The purpose of this report is to describe the e-cohorts we have created, the record-linkages that have been made, and the variables that are available within the datasets to investigate the above research questions.

Cohort description

This programme of research was approved by Scotland's Public Benefit and Privacy Panel for Health (reference: 1819-0051), Scotland's Statistics Public Benefit and Privacy Panel (1819-0051), and the University of Glasgow's College of Medical, Veterinary, and Life Sciences Ethical Committee (reference: 200180081). Data sharing agreements are in place with the data controllers of all the linked datasets.

Table 1 shows the data sources which were linked, the dates of the data collection, the intended research purposes for the data, and the data controllers.

- Insert table 1 about here -

The variables we selected to include from each of the data sources were outcomes, exposures, or potential confounders. A table of the full list of variables is included in supplementary file 1. Data linkage of Scotland's Census, 2011, and de-identification, were carried out by the National Records of Scotland (NRS) Indexing Service (a Trusted Third Party Linkage Service) to the NRS Indexing Spine, which includes each person's Community Health Index. The Community Health Index is the unique NHS identifier given to everyone in Scotland; hence this indexing then enabled linkage to all the other selected health databases. Supplementary file 2 shows the process. The linked datasets are held in Scotland's national safe-haven, where access is provided to the approved members of our team.

Scotland's Census, 2011 includes information on people staying in Scotland on 27 March 2011. It includes people in both private households and communal establishments (e.g. care homes). One householder on behalf of all occupants in private households, or the manager on behalf of all occupants of communal dwellings, was required to complete the Census questionnaire. Non-responders were followed up, and help was provided to complete the form if needed. It was a legal requirement to complete the Census questionnaire, and a statement on the form said that non-completion, or supplying false information could attract a £1,000 fine. It was estimated to have achieved a 94% response rate.[39] During the original data processing, the Census team adjusted for the 6% for whom data were missing, using a Census Coverage Survey (including around 40,000 households) to estimate numbers and characteristics. Self/proxy-reporting was used to identify people with intellectual disabilities, autistic people, and people with other long-term conditions from the Census question 20: 'Do you have any of the following conditions which have lasted, or are expected to last, at least 12 months? Tick all that apply'. Respondents were given a choice of 10 response options: (1) deafness or partial hearing loss, (2) blindness or partial sight loss, (3) learning disability (for example, Down's syndrome), (4) learning difficulty (for example, dyslexia), (5) developmental disorder (for example, autistic spectrum disorder or Asperger's syndrome), (6) physical disability, (7) mental health condition, (8) long-term illness, disease or condition (9) other condition, (10) no condition. Cognitive question testing with retrospective probing was undertaken on question 20 prior to the Census, to test whether the questions were answered accurately and willingly by respondents, and to identify what changes might be required to improve data quality and/or the acceptability of the response options. The pilot testing was

For peer review only - http://bmjopen.bmj.com/site/about/guidelines.xhtml

conducted with 102 participants with a mix of gender and age, both with and without health conditions/disabilities (including people with more than one of the conditions), and included people with autism, intellectual disabilities, dyslexia, dyspraxia, speech impairment, mental health conditions (both milder and more serious), and other long-term conditions. This resulted in a redesign of the question on autism, to the version included in the actual Census, to accurately capture the data on autism, in keeping with feedback from the pilot testing with people with autism. (Henceforth, throughout this paper we refer to "autism", rather than the exact wording on the census described above). Importantly, question 20 distinguished autism from learning disability (which in the UK is synonymous to the international term 'intellectual disabilities'), learning difficulty (which in the UK is synonymous to the international term 'specific learning disability' such as dyslexia), and mental health conditions. All people with a positive response to question 20 on intellectual disabilities and/or autism were included in our record-linked datasets.

The data sources linked to the variables from Scotland's Census, 2011 are described below. All provide routinely collected data for the whole of Scotland. The Scottish Morbidity Record (SMR) routinely records information from episodes of health care.

1. SMR 01 provides data on general/acute hospital admissions and causes using the international statistical classification of diseases and related health problems, 10th edition (ICD-10) codes,[40]
2. SMR 04 provides data on mental health admissions and causes also using ICD-10 codes,
3. SMR 06 is the Cancer Registry and provides information on the disease, its treatments, and outcomes.
4. Prescribing Information System provides information on all medicines that are prescribed and dispensed in the community in Scotland, or issued in hospitals and dispensed in the community, and prescriptions written in Scotland that were dispensed elsewhere in the UK, using British National Formulary codes.
5. Scottish Care Information–Diabetes Collaboration is a national diabetes register that collects data on the quality of care, and outcomes for all patients with diabetes.
6. The national bowel, and cervical screening programmes collect data on screening uptake.
7. Scotland's national death register collates information from death certificates including cause of death as recorded by the certifying clinician, using ICD-10 codes.

Table 2 reports the characteristics of the cohorts with intellectual disabilities, and autism, and the random samples of the general population with neither of these conditions.

- Insert table 2 about here –

Patient and public involvement

This resource and the programme of research it will support, was designed to respond to the growing concern expressed by people with intellectual disabilities, autistic people, their families, and third sector organisations about premature deaths, and poor health-care. The Scottish Learning Disabilities Observatory, where this research is being undertaken, has a specific remit for such work. Its steering group includes partners from third sector organisations, including Down syndrome Scotland, and people with intellectual disabilities, who approved the work plan for this project prior to it commencing.

Findings to date

For peer review only - http://bmjopen.bmj.com/site/about/guidelines.xhtml

To date, research has been completed on the cross-sectional Scottish Census, 2011 data only. We have analysed, compared with the general population, the independent contributions of having intellectual disabilities, and being autistic, on general health. We have also reported general health comparisons between the general population, with autistic people who also have intellectual disabilities. Further, we have reported mental health data compared with the general population on the people with intellectual disabilities (whether or not they were also autistic); autistic people (whether or not they also had intellectual disabilities); and autistic people who also have intellectual disabilities. We then reported on visual and hearing impairments, and physical disabilities in autistic people (whether or not they also had intellectual disabilities); and in autistic people who also had intellectual disabilities. These distinctions are important given the high proportion of overlap between intellectual disabilities and autism.

We derived a dichotomised variable of poor general health from the five response general health question in Scotland's census, 2011. In comparison with the general population, in children/youth, both intellectual disabilities [Odds ratio: OR=18.34 (917.17, 19.58)], and autism [OR=8.40 (8.02, 8.80)] were independently associated with poor general health. In adults, both intellectual disabilities [OR=7.54 (7.02, 8.10)], and autism [OR=4.46 (4.06, 4.89)] were also independently associated with poor general health.[41] People with co-occurring intellectual disabilities and autism combined were particularly vulnerable to poor general health.[42]

In comparison with the general population, mental health conditions (answered as "yes" or "no") were also more common in people with intellectual disabilities [OR=7.1 (6.8, 7.3)],[43] autistic adults [OR=8.6 (8.2, 9.0)],[44] and people with co-occurring intellectual disabilities and autism [OR=130.8 (117.1, 146.1)].[45] Poor general health was also associated with poor mental health in people with intellectual disabilities.[43]

Visual impairment, hearing impairment, and physical disability were also more common than in the general population. For autistic adults, and autistic children/youth respectively, visual impairment [OR=8.5 (7.9, 9.2) and OR=8.9 (8.1-9.7)]; hearing impairment [OR=3.3 (3.1, 3.6) and OR=5.4 (5.1-5.6)]; and physical disability [OR=6.2 (5.8, 6.6) and OR=15.8 (14.1-17.8)] were considerably more common.[44,46] For people with both intellectual disabilities and autism, visual impairment [OR=65.9 (95% CI 58.7, 73.9)]; hearing impairment [OR=22.0 (95% CI 19.2, 25.2)]; and physical disability [OR=157.5 (95%=CI 144.6, 171.7)] were even more common.[44]

Raising awareness of these issues is important, as these conditions are disabling, and in some cases life-limiting if left untreated. In addition to the communication needs that people with intellectual disabilities and autistic people have due to their neurodevelopmental condition, comorbidity renders differential diagnosis and treatments more complex, particularly if unrecognised, hence it is essential that clinicians are aware of the additional conditions that their patients may have.

Strengths and limitations

Strengths of the e-cohorts include their large scale, national Census coverage of the whole country of Scotland, with 94% uptake, and a high level of successful record linkage (>92%), hence limiting bias, and the systematic enquiry about intellectual disabilities and autism of the whole population. The questions on intellectual disabilities and autism were subject to cognitive question testing prior to use, to ensure that they accurately captured the conditions,

For peer review only - http://bmjopen.bmj.com/site/about/guidelines.xhtml

and were acceptable to the population. Additionally, specific learning disabilities were distinguished from intellectual disabilities or autism, which is important given lay terms in current use e.g. in the UK and Republic of Ireland. We are not aware of any other national Census data which has included data on these conditions, other than Irish data which has the limitation of not distinguishing intellectual disabilities from specific learning disabilities. Scotland has a rich profile of routinely collected health data which has been successfully used many times in previous research on the general population, and linkage of Scotland's Census, 2011 to these data, has provided large scale, whole country cohorts of intellectual disabilities, and of autism.

Limitations include that routinely collected data lack disease-specificity and granularity, and cause of death data is infrequently verified by post-mortem. Ascertainment of intellectual disabilities and autism was based on self or proxy reporting. Scotland is a high income country with good country-wide educational and diagnostic services, and diagnosis of neurodevelopmental conditions attracts additional support for learning so is advantageous for the child. If a child's learning is delayed, schools have a statutory responsibility to seek assessment, so such children will have received a formal diagnosis. The families of children with learning delay are integral to the assessment and diagnostic care pathway for the child, and the development of the co-ordinated support plan for their additional support needs. Proxy-reporting is the basis for much of the healthcare provided for people with intellectual disabilities and autistic people who cannot self-report, and overall, proxy reports are concluded to be a useful addition to determine aspects of wellbeing when the need arises.[47] Those adults for whom a diagnosis of autism was recorded will reflect the contemporary diagnostic practices in place during their childhood; and the concept of the autism spectrum has broadened in recent years.

Collaboration

The breadth of the datasets created means they are suitable to be repurposed to answer many additional questions to the ones we outline in our current programme of research. Collaboration is encouraged, and as we are not the data controllers, this would be subject to approval by Scotland's Public Benefit and Privacy Panel for Health, Scotland's Statistics Public Benefit and Privacy Panel, and ethical approval by an appropriate ethical committee.

References

1. O'Leary L, Hughes-McCormack L, Cooper S-A. Early death and causes of death of people with intellectual disabilities: A systematic review. *J Appl Res Intellect Disabil* 2017;31:325-42.
2. O'Leary L, Hughes-McCormack L, Cooper S-A. Life expectancy and causes of death of people with Down syndrome: a systematic review. *J Appl Res Intellect Disabil* 2018;31:687-708.
3. Glover G, Williams R, Heslop P, *et al.* Mortality in people with intellectual disabilities in England. *J Intellect Disabil Res* 2017;61:62-74.
4. Cooper S-A, Allan L, Greenlaw, N, *et al.* Rates, causes, place, and predictors of death in adults with intellectual disabilities with and without Down syndrome: prospective cohort study with record linkage. *BMJ Open* 2020;10:e036465.
5. Smith GS, Fleming M, Kinnear D, *et al.* Rates and causes of mortality among children and young people with and without intellectual disabilities in Scotland: a record linkage cohort study of 796,190 schoolchildren. *BMJ Open* 2020;P10e034077:doi: 10.1136/bmjopen-2019-034077

For peer review only - http://bmjopen.bmj.com/site/about/guidelines.xhtml

6. Mouridsen SE, Brønnum-Hansen H, Rich B, Isager T. Mortality and causes of death in autism spectrum disorders: an update. *Autism* 2008;12:403-14.
7. Hirvikoski T, Mittendorfer-Rutz E, Boman M, Larsson H, Lichtenstein P, Bölte S. Premature mortality in autism spectrum disorder. *Br J Psychiatry* 2016;208:232-238.
8. Hwang YI, Srasuebkul P, Foley KR, Arnold S, Troller JN. Mortality and cause of death of Australians on the autism spectrum. *Autism Res* 2019;12:9806-815.
9. Smith GS, Fleming M, Kinnear D, *et al.* Mortality in 787,666 school pupils in Scotland with and without autism: a cohort study. *Autism* 2021;25:300–304.
10. Heslop P, Blair PS, Fleming P, *et al.* The Confidential Inquiry into premature deaths of people with intellectual disabilities in the UK: a population-based study. *Lancet* 2014;383:889–95.
11. Hosking FJ, Carey IM, Shah SM, *et al.* Mortality among adults with intellectual disability in England: Comparisons with the general population. *Am J Public Healt,* 2016;106:1483–90. ERRATUM: *Am J Public Health* 2016;106:e11–e12. doi: 10.2105/AJPH.2016.303240e
12. Cooper S-A, Hughes-McCormack L, Greenlaw N, *et al.* Management and prevalence of long-term conditions in primary health care for adults with intellectual disabilities compared with the general population: a population-based cohort study. *J Appl Res Intellect Disabil* 2017;31(S1):68–81. 10.1111/jar.12386
13. Kinnear D, Morrison J, Allan L, *et al.* The prevalence of multi-morbidity in a cohort of adults with intellectual disabilities, with and without Down syndrome. *BMJOpen* 2018; 0:e018292.
14. Cooper S-A, McLean G, Guthrie B, *et al.* Multiple physical and mental health comorbidity in adults with intellectual disabilities: population-based cross-sectional analysis. *BMC Family Practice* 2015;DOI:10.1186/s12875-015-0329-3
15. Lunsky Y, Lin E, Balogh R, Klein-Geltink J. Diabetes prevalence among persons with serious mental illness and developmental disability. *Psychiatric Service* 2011;62:830.
16. McVilly K, McGillivray J, Curtis A, Lehman J, Morrish L, Speight J. Diabetes in people with an intellectual disability: a systematic review of prevalence, incidence and impact. *Diabetic Med* 2014;31:897-904.
17. Tyrer F, Dunkley AJ, Singh J. *et al*. Multimorbidity and lifestyle factors among adults with learning disability: a cross-sectional analysis of a UK cohort. *J Intellect Disabil Res* 2019;63:255-265.
18. Melville CA, McGarty A, Harris L, *et al.* A population based study of the prevalence and correlates of sedentary behaviour of adults with intellectual disabilities. *J Intellect Disabil Res* 2018;62:60-71.
19. Sullivan SG, Hussain R, Threlfall T, Bittles AH. The incidence of cancer in people with intellectual disabilities. *Cancer Causes & Control* 2004;15:1021-1025.
20. Patja K, Eero P, Iivanainen M. Cancer incidence among people with intellectual disability. *J Intellect Disabil Res* 2001;45:300-7.
21. Böhmer CJM, Klinkenberg-Knol EC, Niezen-de-Boer RC, Meuwissen SG. The age-related incidence of oesophageal carcinoma in intellectually disabled individuals in institutions in the Netherlands. *European J Gastroenterology and Hepatology* 1997;9:589-92.
22. Hughes-McCormack LA, Rydzewska E, Henderson A, *et al.* Prevalence and general health status of people with intellectual disabilities in Scotland -a total population study. *J Epidemiology Community Health* 2017;72:78–85.
23. Rydzewska E, Hughes-McCormack LA, Gillberg C, *et al.* General health of adults with autism spectrum disorder -a whole country population cross-sectional study. *Res Autism Spectrum Disorders* 2019;60:59-66.

For peer review only - http://bmjopen.bmj.com/site/about/guidelines.xhtml

24. Rydzewska E, Hughes-McCormack LA, Gillberg C, *et al.* Age at identification, prevalence and general health of children with autism: observational study of a whole country population. *BMJOpen* 2019;9:e025904.
25. Rydzewska E, Dunn K, Cooper S-A. An umbrella systematic review on systematic reviews and meta-analyses on comorbid health conditions in people with autism spectrum disorder. *Br J Psychiatry* 2021;218:10-19.
26. Ali A, Scior K, Ratti V, Strydom A, King M, Hassiotis A. Discrimination and other barriers to accessing health care: perspectives of patients with mild and moderate intellectual disability and their carers. *PLoS ONE* 2013;8(8):e70855. https://doi.org/10.1371/journal.pone. 0070855
27. Calleja S, Islam FMA, Ingsley J, McDonald R. Healthcare access for autistic adults. A systematic review. *Med* 2020;99(29):e20899
28. Cobigo V, Oullette-Kuntz H, Balogh R, Leung F, Lin E, Lunsky Y. Are cervical and breast cancer screening programmes equitable? The case of women with intellectual disabilities and developmental disabilities. *J Intellect Disabil Res* 2013;57:478–488.
29. Osborn DPJ, Horsfall L, Hassiotis A, Petersen I, Walters K, Nazareth I. Access to cancer screening in people with learning disabilities in the UK: cohort study in the health improvement network, a primary care research database. *PLoS ONE* 2012;7(8):e43841. DOI: 10.1371/journal.pone.0043841
30. Dunn K, Hughes-McCormack L, Cooper S-A. Hospital admissions for physical health conditions for people with intellectual disabilities: Systematic Review. *J App Res Intellect Disabil* 2017;31 (S1):1–10. DOI: 10.1111/jar.12360
31. Hand BN, Boan AD, Bradley CC, Charle, JM, Carpenter LA. Ambulatory care sensitive admissions in individuals with autism spectrum disorder, intellectual disability, and population controls. *Autism Res* 2019;12:295-302.
32. Balogh R, Leonard H, Bourke J, *et al.* Data Linkage: Canadian and Australian Perspectives on a Valuable Methodology for Intellectual and Developmental Disability Research. *Intellect Dev Disabil* 2019;57(5):439-462.
33. Reppermund S, Srasuebkul P, Heintze T, *et al.* Cohort profile: a data linkage cohort to examine health service profiles of people with intellectual disability in New South Wales, Australia. BMJ Open 2017;7: e015627.
34. Cooper S-A, Smiley E, Morrison J, *et al.* Prevalence of and associations with mental ill-health in adults with intellectual disabilities. *Br J Psychiatry,* 2007;190:27-35.
35. Havercamp SM, Krahn GL. What matters in population health and how we count it among people with intellectual and developmental disabilities. *Intellect Dev Disabil* 2019;57:347-356.
36. Wadsworth ME, Mann SL, Rodgers B, Kuh DJ, Hilder WS, Yusef EJ. (1992) Loss and representativeness in a 43 year follow up of a national birth cohort. *J Epidemiol Community Health* 1992;46:300-304.
37. Gould J, Ashton-Smith J. Missed diagnosis or misdiagnosis: girls and women on the autism spectrum. *Good Autism Practice* 2011;12(1):34-41.
38. Brugha TS, Spiers N, Bankart J, *et al.* Epidemiology of autism in adults across age groups and ability levels. *Br J Psychiatry,* 2016;209:498-503.
39. National Records of Scotland (NRS). *2011 Census Release 1C - How the 2011 Census population estimates were obtained*. Edinburgh: National Records of Scotland 2013.
40. World Health Organisation. *International Statistical Classification of Diseases and Related Health Problems, 10th Revision.* Geneva: World Health Organisation, 1992.
41. Kinnear D, Rydzewska E, Dunn K, *et al.* The relative influence of intellectual disabilities and autism on mental and general health. A cross-sectional study of a whole country of 5.3 million children and adults. *BMJ Open* 2019;9(8):e029040.

For peer review only - http://bmjopen.bmj.com/site/about/guidelines.xhtml

42. Dunn KD, Rydzewska E, Rintoul J, Macintyre C, Cooper S-A. The prevalence and general health status of people with intellectual disabilities and autism co-occurring together: a total population study. *J Intellect Disabil Res* 2019;63:277-285.
43. Hughes-McCormack L, Rydzewska E, Henderson A, Rintoul J, Macintyre C, Cooper S-A. Prevalence of mental ill-health and relationship with physical health in a whole country population of 26,349 people with intellectual disabilities, compared with the general population. *Br J Psychiatry Open* 2017;3,Issue 5:http://bjpo.rcpsych.org/content/3/5/243
44. Rydzewska E, Hughes-McCormack LA, Gillberg C, *et al.* Prevalence of long-term health conditions in adults with autism - observational study of a whole country population. *BMJOpen* 2018;8:e023945.
45. Dunn K, Rydzewska E, Cooper S-A. The prevalence of mental health conditions, sensory impairments, and physical disability in people with co-occurring intellectual disabilities and autism compared with other people – a total population study of 5,295,403 people. *BMJ Open* 2020;10:e035280.
46. Rydzewska E, Hughes-McCormack LA, Gillberg C, *et al.* Prevalence of sensory impairments, physical and intellectual disabilities, and mental health in children and young people with self/proxy-reported autism - observational study of a whole country population. *Autism* 2019;23(5):1201-1209.
47. Perkins EA. Self- and proxy reports across three populations: older adults, persons with Alzheimer's disease, and persons with intellectual disabilities. *J Policy Practice Intellect Disabil* 2007;4:1-10.

Funding declaration

This work was supported by the UK Medical Research Council, grant number: MC_PC_17217), the Scottish Government via the Scottish Learning Disabilities Observatory, and the Baily Thomas charitable fund.

The study sponsor and funders had no role in the study design; in the collection, analysis, and interpretation of data; in the writing of the report; and in the decision to submit the article for publication.

The researchers are independent from the funders.

Data sharing statement

Data access is available through approval by Scotland's Public Benefit and Privacy Panel for Health, Scotland's Statistics Public Benefit and Privacy Panel, and an appropriate ethical committee.

Competing interests

The authors declare no competing interests.

Contributor statement

S-AC conceived and designed the programme, and wrote the first draft of the manuscript. AH and DK contributed to the design, and project management. DMcK, MF, and GS designed and supervised the statistics. LH-McC, ER, and KD analysed data. JP contributed expertise

For peer review only - http://bmjopen.bmj.com/site/about/guidelines.xhtml

on data linkage and datasets. CM oversees programme management. All contributed to drafting the manuscript, and approved the final version. S-AC is the guarantor.

Acknowledgements
The authors would like to acknowledge the support of the eDRIS Team (Public Health Scotland), in particular David Clark, for their involvement in obtaining approvals, provisioning and linking data, and for the use of the secure analytical platform within the National Safe Haven.

Word count
3,618

For peer review only

Table 1. Linked data sources included in the two datasets

DATASET 1

Resource	Time period	Use	Data controller
Scotland's Census, 2011	27.3.11	To identify the populations with intellectual disabilities, and autism, and the comparison population, and their basic demography	National Records of Scotland
Register of Deaths	27.3.11-15.08.20	To identify deaths, causes of deaths, and treatable deaths	National Records of Scotland
Scottish Morbidity Records 06	27.3.11-15.08.20	To identify cancer incidence, mortality, and treatments (quality of health care)	NHS National Services Scotland, Information Services Division

DATASET 2

Resource	Time period	Use	Data controller
Scotland's Census, 2011	27.3.11	To identify the populations with intellectual disabilities, and autism, and the comparison population, their basic demography, household, employment, and long-term conditions data (potential determinants of mortality)	National Records of Scotland
Register of Deaths	27.3.11-15.08.20	To identify deaths	National Records of Scotland
Prescribing Information System	27.3.11-27.9.11	To identify people with: asthma/COPD; angina/congestive heart failure/hypertension; peptic ulcer/reflux; constipation; diabetes; thyroid disorder; depression; bipolar disorders; anxiety/sleep; psychosis; ADHD; epilepsy; glaucoma (health burden)	NHS National Services Scotland, Information Services Division
Scottish Morbidity Records 01	27.3.11-15.08.20	To identify general/acute hospital admissions and causes, and ambulatory care sensitive admissions	NHS National Services Scotland, Information Services Division
Scottish Morbidity Records 04	27.3.11-15.08.20	To identify mental health admissions and their causes	NHS National Services Scotland, Information Services Division
SCI-diabetes	27.3.11-15.08.20	To identify quality of diabetic care and diabetic outcomes (quality of health care)	Caldicott guardians
National Bowel Screening Programme	27.3.11-15.08.20	To identify screening uptake	NHS National Services Scotland, Information Services Division
Cervical screening	2016-15.08.20	To identify screening uptake	NHS National Services Scotland, Information Services Division

For peer review only - http://bmjopen.bmj.com/site/about/guidelines.xhtml

Table 2. Characteristics of the cohorts

Variable	Category	Dataset 1 and 2 Intellectual disabilities N=22,538 (100%)	Dataset 1 and 2 Autism N=27,741 (100%)	Dataset 1 15% of general population† N=700,437 (100%)	Dataset 2 5% of general population† N=233,378 (100%)
**Sex**	Male	12,837 (57.0%)	21,390 (77.1%)	335,226 (47.9%)	111,497 (47.8%)
	Female	9,701 (43.0%)	6,351 (22.9%)	365,211 (52.1%)	121,881 (52.2%)
**Age**	0-15	4,509 (20.0%)	15,387 (55.5%)	117,980 (16.8%)	39,220 (16.8%)
	15-24	3,546 (15.7%)	6,790 (24.5%)	76,184 (10.9%)	25,574 (11.0%)
	25-34	2,976 (13.2%)	2,024 (7.3%)	83,868 (12.0%)	27,721 (11.8%)
	35-45	3,277 (14.5%)	1,300 (4.7%)	97,886 (14.0%)	32,599 (14.0%)
	45-54	3,664 (16.3%)	1,053 (3.8%)	108,052 (15.4%)	36,164 (15.5%)
	55-64	2,494 (11.1%)	617 (2.2%)	92,698 (13.2%)	31,022 (13.3%)
	65-74	1,330 (5.9%)	300 (1.1%)	67,532 (9.6%)	22,166 (9.5%)
	75+	742 (3.3%)	270 (1.0%)	56,237 (8.0%)	18,912 (8.1%)
**Ethnicity***	White	22,022 (97.7%)	26,976 (97.2%)	Not available	225,626 (96.7%)
	Asian/Asian Scottish/Asian British	368 (1.6%)	425 (1.5%)	Not available	5,181 (2.2%)
	African	35 (0.2%)	80 (0.3%)	Not available	928 (0.4%)
	Caribbean or black	21 (0.1%)	24 (0.1%)	Not available	213 (0.1%)
	Mixed/multiple ethnic groups	66 (0.3%)	189 (0.7%)	Not available	888 (0.4%)
	Other ethnic group	26 (0.1%)	47 (0.2%)	Not available	542 (0.2%)
**SIMD**	1 – most deprived	6,200 (27.5%)	6,748 (24.3%)	130,354 (18.6%)	42,828 (18.4%)
	2	5,481 (24.3%)	5,901 (21.3%)	135,311 (19.3%)	45,407 (19.5%)
	3	4,516 (20.0%)	5,678 (20.5%)	142,238 (20.3%)	47,886 (20.5%)
	4	3,693 (16.4%)	5,128 (18.5%)	147,866 (21.1%)	49,429 (21.2%)
	5 – most affluent	2,648 (11.7%)	4,286 (15.5%)	144,668 (20.7%)	47,828 (20.5%)
**General health status***	Very good	3,389 (15.0%)	10,510 (38.0%)	Not available	122,511 (52.4%)
	Good	7,942 (35.2%)	9,608 (34.6%)	Not available	69,575 (30.0%)
	Fair	7,715 (34.2%)	5,387 (19.4%)	Not available	28,273 (12.1%)
	Bad	2,356 (10.5%)	1,533 (5.5%)	Not available	9,885 (4.2%)
	Very bad	1,136 (5.0%)	703 (2.5%)	Not available	3,134 (1.3%)
**Mental health condition***	Yes	4,755 (21.1%)	3,998 (14.4%)	Not available	9,710 (4.2%)
	No	17,783 (78.9%)	23,743 (85.6%)	Not available	223,668 (95.8%)

SIMD=Scottish Index of Multiple Deprivation
†General population with neither intellectual disabilities nor autism
*Data not available for Dataset 1 to ensure data minimisation, as it does not have sufficient power to investigate these factors in relation to causes of deaths and cancers

For peer review only - http://bmjopen.bmj.com/site/about/guidelines.xhtml

Figure. Example power calculations across comparison sample sizes

For peer review only

For peer review only - http://bmjopen.bmj.com/site/about/guidelines.xhtml

Females only

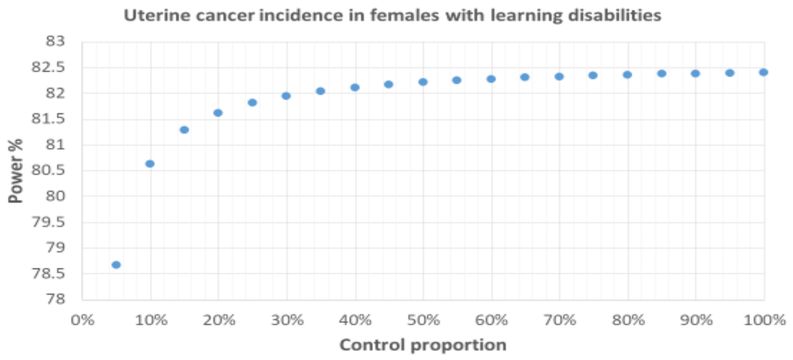

- Proportion of control population - 5% (n=135,838) to 100% n=(2,716,759)
- Based on uterine cancer incidence rate of 29.1 cases per 100,000 in the female general population
- Standardised mortality ratio of 2.98 (Sullivan et al. 2004) in 11,200 females with learning disabilities

Males only

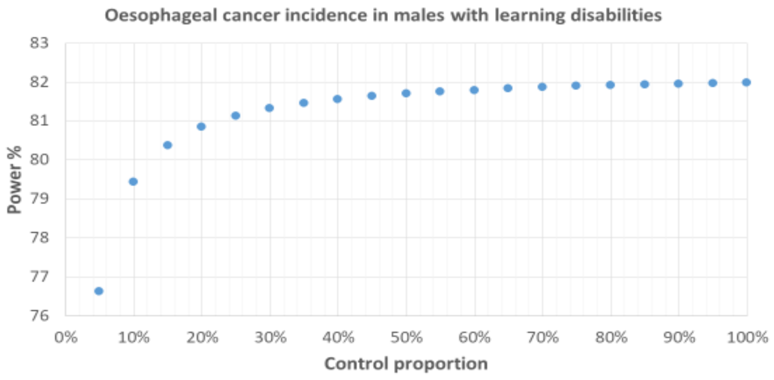

- Proportion of control population –5% (n=127,615) to 100% (n=2,552,295)
- Based on oesophageal cancer incidence rate of 22.8 cases per 100,000 in general population males
- Standardised mortality ratio of 2.9 (Böhmer et al. 1997) in 15,149 males with learning disabilities

Males only

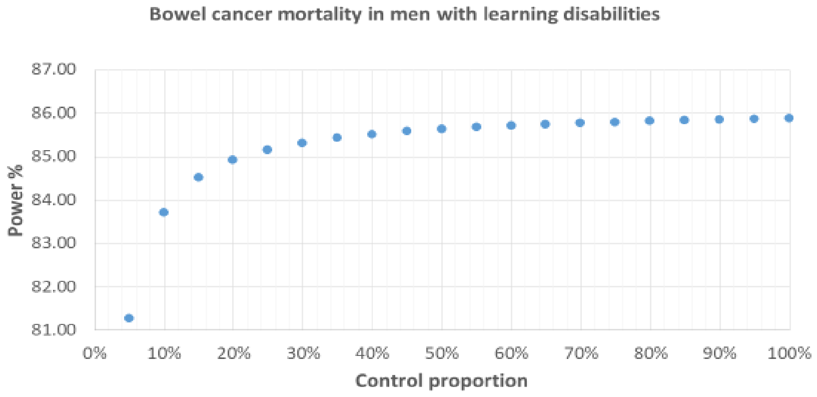

- Proportion of control population –5% (n=127,615) to 100% (n=2,552,295)
- Based on annual Scottish cancer mortality rate of 32.6 deaths per 100,000
- Standardised mortality ratio of 2.68 (Glover et al. 2015) in 15,149 males with learning disabilities

Supplementary file 1. List of variables included in the two datasets

DATASET 1

Type of variable	Outcome	Exposure	Potential confounding factors
**Scotland's Census 2011**			
Read-through Index number for linkage		N/A	N/A
Month and year of birth			X
Living arrangement - seven exclusive values			X
Scottish Index of Multiple Deprivation 2012			X
Urban-rural classification 8-fold 2011-12			X
Heath Board area 2011			X
Council area 2011			X
Long-term health condition: autism		X	
Long-term health condition: learning disability		X	
Sex			X
**NRS Death register**			
CHI		N/A	N/A
Age at death	X		
Main cause of death	X		
All causes of death (1-11)	X		
Date of death	X		
Scottish Index of Multiple Deprivation 2012			X
Place of death			X
**SMR06**			
CHI		N/A	N/A
Date of incidence	X		
Date of registration	X		
Death certificate only	X		
Side			X
Site (ICD09/ICD10/ICD02)	X		
Type ICD0/ICD02/ICD03	X		
Treated with surgery			X
Treated with radiotherapy			X
Treated with chemotherapy			X
Treated with hormones			X
Treated with other therapy			X

For peer review only

DATASET 2

Type of variable	Outcome	Exposure	Potential confounding factors
**Scotland's Census 2011**			
Read-through Index number for Census 2011		N/A	N/A
Month and year of birth			X
Living arrangements - seven exclusive values			X
Scottish Index of Multiple Deprivation 2012			X
Urban-rural classification 8-fold 2011-12			X
Heath Board area 2011			X
Council area 2011			X
Activity last week			X
Adults in employment, number in household			X
Adults in household, number of			X
Carers in household with economic activity			X
Provision of unpaid care			X
Cars or vans, number of			X
Central heating type in household			X
Unpaid carers in household, number of			X
Limitation of activities			X
Distance travelled to work or place of study			X
Recorded ethnicity			X
Nature of communal establishment			X
Ever employed			X
Number of residents in family			X
Family status			X
Count of dependent children in family			X
General Health status			X
Highest level of qualification			X
Hours worked (grouped)			X
Language proficiency			X
Marital status			X
Long-term health condition: blind		X	X
Long-term health condition: autism		X	X
Long-term health condition: deaf		X	X
Long-term health condition: dyslexia		X	X
Long-term health condition: learning disability		X	X
Long-term health condition: mental health condition		X	X
Long-term health condition: physical disability		X	X

For peer review only

Type of variable	Outcome	Exposure	Potential confounding factors
Occupancy rating			X
Occupancy status			X
Parent to household member			X
Population density of datazone			X
Persons per room, number in household			X
Sex			X
Household size			X
Students living away from home during term time (aged 4 and over)			X
Schoolchild or full-time student indicator			X
Tenure of household			X
Term-time address indicator			X
Accommodation type			X
**NRS Death register**			
CHI		N/A	N/A
Age at death	X		
Main cause of death	X		
All causes of death (1-11)	X		
Month of death	X		
Scottish Index of Multiple Deprivation 2012			X
Place of death			X
**Prescribing Information System**			
CHI		N/A	N/A
Date medication prescribed			X
Date medication dispensed			X
Date medication paid			X
BNF codes of drugs for: asthma/COPD; angina/congestive heart failure/hypertension; peptic ulcer/reflux; constipation; diabetes; thyroid dysfunction; depression; bipolar disorder; anxiety/sleep; psychosis; ADHD; epilepsy; glaucoma		X	X
BNF root drug		X	
Prescribed item name/approved name		X	
Prescribed item strength			X
Number of defined daily doses dispensed			X
Number of dispensed/paid items			X
Dispensed/paid quantity			X
**SMR01**			
CHI		N/A	N/A
Date of admission	X		
Date of discharge	X		
Main condition on discharge	X		

For peer review only

Type of variable	Outcome	Exposure	Potential confounding factors
Secondary condition on discharge (1-5)	X		
Main operation	X		
Other operations (1-3)	X		
Length of stay	X		
CIS marker	X		
Inpatient/day case marker	X		
Type of facility/management patient			X
Type of admission	X		
Admission reason	X		
**SMR04**			
CHI		N/A	N/A
Date of admission	X		
Date of discharge	X		
Main condition on discharge	X		
Secondary condition on discharge (1-5)	X		
Main operation	X		
Other operations (1-3)	X		
Main condition on admission	X		
Other conditions on admission (1-3)	X		
Previous psychiatric care			X
Type of psychiatric care			X
**SCI-Diabetes**			
CHI		N/A	N/A
Date of diagnosis			X
Type of diabetes		X	
HbA1c	X		
Hypoglycaemic coma	X		
Attendance at hospital diabetes clinic	X		
Arrangements for formal diabetes care	X		
Drugs			X
Date of death	X		
Cause of death	X		
Underlying cause of death	X		
Weight			X
Height			X
Body Mass Index			X
Systolic bp			X
Diastolic bp			X
Smoking status at date of contact			X
Year patient stopped smoking			X
Alcohol intake			X
Diabetic review on this date	X		
Referral to defined health care professional	X		
Seen by defined health care professional	X		

For peer review only

Type of variable	Outcome	Exposure	Potential confounding factors
**National Bowel Screening programme**			
CHI		N/A	N/A
Health board of residence			X
Date screening test kit sent			X
Screening test result	X		
Colonoscopy performed?	X		
Reason for not having a colonoscopy	X		
Colonoscopy completed	X		
Invasive cancer detected?	X		
ICD 10 classification of neoplasm	X		
Complication arising from colonoscopy			X
TNM derived Dukes' stage	X		
Polyps detected	X		
Adenoma detected	X		
**Cervical Cancer screening**			
CHI		N/A	N/A
Date of test result	X		
SIMD 2012 deprivation category			X
Date of HPV Immunisation	X		
HPV Immunisation Status	X		
Screened within 3.5yr	X		
Screened within 5.5yr	X		

For peer review only

Supplementary file 2. Data processing flowchart

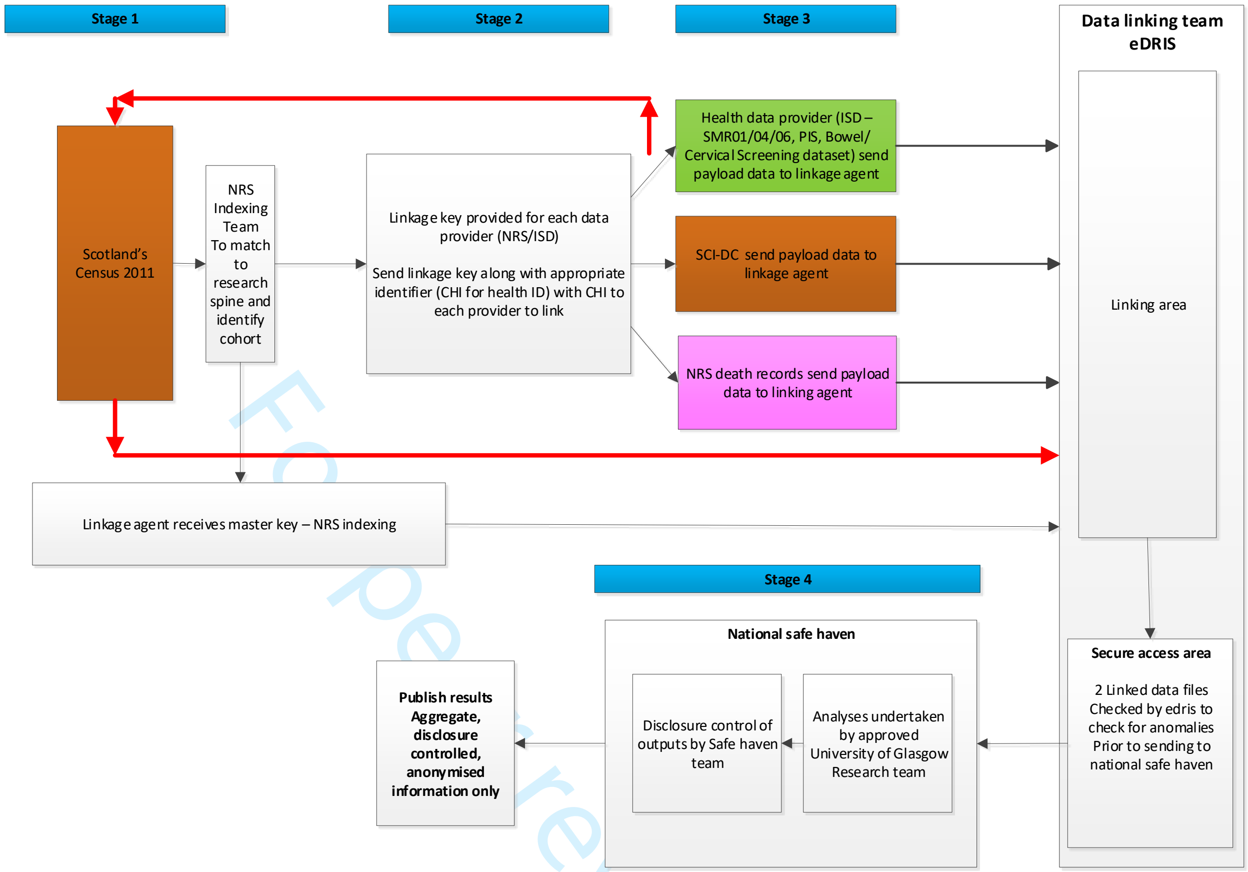

CHI=community health index; eDRIS=electronic data research and innovation service; ISD=information services division of national services Scotland; NRS=national records of Scotland; PIS=prescribing information system; SCI-DC=Scottish care information–diabetes collaboration; SMR=Scottish morbidity record

STROBE (Strengthening The Reporting of OBservational Studies in Epidemiology) Checklist

A checklist of items that should be included in reports of observational studies. You must report the page number in your manuscript where you consider each of the items listed in this checklist. If you have not included this information, either revise your manuscript accordingly before submitting or note N/A.

Note: An Explanation and Elaboration article discusses each checklist item and gives methodological background and published examples of transparent reporting. The STROBE checklist is best used in conjunction with this article (freely available on the Web sites of PLoS Medicine at http://www.plosmedicine.org/, Annals of Internal Medicine at http://www.annals.org/, and Epidemiology at http://www.epidem.com/). Information on the STROBE Initiative is available at www.strobe-statement.org.

Section and Item	Item No.	Recommendation	Reported on Page No.
**Title and Abstract**	1	(*a*) Indicate the study's design with a commonly used term in the title or the abstract	
		(*b*) Provide in the abstract an informative and balanced summary of what was done and what was found	
**Introduction**			
Background/Rationale	2	Explain the scientific background and rationale for the investigation being reported	
Objectives	3	State specific objectives, including any prespecified hypotheses	
**Methods**			
Study Design	4	Present key elements of study design early in the paper	
Setting	5	Describe the setting, locations, and relevant dates, including periods of recruitment, exposure, follow-up, and data collection	
Participants	6	(*a*) *Cohort study*—Give the eligibility criteria, and the sources and methods of selection of participants. Describe methods of follow-up<br>*Case-control study*—Give the eligibility criteria, and the sources and methods of case ascertainment and control selection. Give the rationale for the choice of cases and controls<br>*Cross-sectional study*—Give the eligibility criteria, and the sources and methods of selection of participants	
		(*b*) *Cohort study*—For matched studies, give matching criteria and number of exposed and unexposed<br>*Case-control study*—For matched studies, give matching criteria and the number of controls per case	
Variables	7	Clearly define all outcomes, exposures, predictors, potential confounders, and effect modifiers. Give diagnostic criteria, if applicable	

For peer review only

Section and Item	Item No.	Recommendation	Reported on Page No.
Data Sources/ Measurement	8*	For each variable of interest, give sources of data and details of methods of assessment (measurement). Describe comparability of assessment methods if there is more than one group	
Bias	9	Describe any efforts to address potential sources of bias	
Study Size	10	Explain how the study size was arrived at	
Quantitative Variables	11	Explain how quantitative variables were handled in the analyses. If applicable, describe which groupings were chosen and why	
Statistical Methods	12	(*a*) Describe all statistical methods, including those used to control for confounding	
		(*b*) Describe any methods used to examine subgroups and interactions	
		(*c*) Explain how missing data were addressed	
		(*d*) *Cohort study*—If applicable, explain how loss to follow-up was addressed<br>*Case-control study*—If applicable, explain how matching of cases and controls was addressed<br>*Cross-sectional study*—If applicable, describe analytical methods taking account of sampling strategy	
		(*e*) Describe any sensitivity analyses	
**Results**			
Participants	13*	(a) Report numbers of individuals at each stage of study—eg numbers potentially eligible, examined for eligibility, confirmed eligible, included in the study, completing follow-up, and analysed	
		(b) Give reasons for non-participation at each stage	
		(c) Consider use of a flow diagram	
Descriptive Data	14*	(a) Give characteristics of study participants (eg demographic, clinical, social) and information on exposures and potential confounders	
		(b) Indicate number of participants with missing data for each variable of interest	
		(c) *Cohort study*—Summarise follow-up time (eg, average and total amount)	
Outcome Data	15*	*Cohort study*—Report numbers of outcome events or summary measures over time	
		Case-control study—Report numbers in each exposure category, or summary measures of exposure	
		Cross-sectional study—Report numbers of outcome events or summary measures	

For peer review only

Section and Item	Item No.	Recommendation	Reported on Page No.
Main Results	16	(*a*) Give unadjusted estimates and, if applicable, confounder-adjusted estimates and their precision (eg, 95% confidence interval). Make clear which confounders were adjusted for and why they were included	
		(*b*) Report category boundaries when continuous variables were categorized	
		(*c*) If relevant, consider translating estimates of relative risk into absolute risk for a meaningful time period	
Other Analyses	17	Report other analyses done—eg analyses of subgroups and interactions, and sensitivity analyses	
**Discussion**			
Key Results	18	Summarise key results with reference to study objectives	
Limitations	19	Discuss limitations of the study, taking into account sources of potential bias or imprecision. Discuss both direction and magnitude of any potential bias	
Interpretation	20	Give a cautious overall interpretation of results considering objectives, limitations, multiplicity of analyses, results from similar studies, and other relevant evidence	
Generalisability	21	Discuss the generalisability (external validity) of the study results	
**Other Information**			
Funding	22	Give the source of funding and the role of the funders for the present study and, if applicable, for the original study on which the present article is based	

*Give information separately for cases and controls in case-control studies and, if applicable, for exposed and unexposed groups in cohort and cross-sectional studies.

Once you have completed this checklist, please save a copy and upload it as part of your submission. DO NOT include this checklist as part of the main manuscript document. It must be uploaded as a separate file.

For peer review only
